# Rapid Whole-Genome Sequencing in Critically Ill Infants and Children with Suspected, Undiagnosed Genetic Diseases: Evolution to a First-Tier Clinical Laboratory Test in the Era of Precision Medicine

**DOI:** 10.3390/children12040429

**Published:** 2025-03-28

**Authors:** Rina Kansal

**Affiliations:** 1 Molecular Oncology and Genetics, Diagnostic Laboratories, Versiti Blood Center of Wisconsin, Milwaukee, WI 53233, USA; rinakansal@msn.com; 2 Department of Pathology and Anatomical Sciences, The University at Buffalo, Buffalo, NY 14260, USA

**Keywords:** exome sequencing, genome sequencing, chromosomal microarray, pediatric genetics, rapid genomic sequencing, neonatology, intensive care, precision medicine, ethics, secondary findings

## Abstract

The completion of the Human Genome Project in 2003 has led to significant advances in patient care in medicine, particularly in diagnosing and managing genetic diseases and cancer. In the realm of genetic diseases, approximately 15% of critically ill infants born in the U.S.A. are diagnosed with genetic disorders, which comprise a significant cause of mortality in neonatal and pediatric intensive care units. The introduction of rapid whole-genome sequencing (rWGS) as a first-tier test in critically ill children with suspected, undiagnosed genetic diseases is a breakthrough in the diagnosis and subsequent clinical management of such infants and older children in intensive care units. Rapid genome sequencing is currently being used clinically in the USA, the UK, the Netherlands, Sweden, and Australia, among other countries. This review is intended for students and clinical practitioners, including non-experts in genetics, for whom it provides a historical background and a chronological review of the relevant published literature for the progression of pediatric diagnostic genomic sequencing leading to the development of pediatric rWGS in critically ill infants and older children with suspected but undiagnosed genetic diseases. Factors that will help to develop rWGS as a clinical test in critically ill infants and the limitations are briefly discussed, including an evaluation of the clinical utility and accessibility of genetic testing, education for parents and providers, cost-effectiveness, ethical challenges, consent issues, secondary findings, data privacy concerns, false-positive and false-negative results, challenges in variant interpretation, costs and reimbursement, the limited availability of genetic counselors, and the development of evidence-based guidelines, which would all need to be addressed to facilitate the implementation of pediatric genomic sequencing in an effective widespread manner in the era of precision medicine.

## 1. Introduction

Rare genetic diseases affect numerous children and young adults. While the number of individuals or families affected by any unique genetic disease might be small, the total number of all individuals affected by all rare genetic diseases is substantial. The incidence and prevalence of genetic diseases in children are challenging to estimate. In 1977, monogenic diseases were estimated to collectively affect about 10 individuals per 1000 live births, including 7/1000 for autosomal-dominant, 2.5/1000 for autosomal-recessive, and 0.4/1000 for X-linked inherited diseases [1]. In 1988, Baird et al. studied more than one million consecutive live births from a Canadian population-based registry. They reported that genetic diseases were expected to occur in ≥53/1000 live-born individuals younger than 25 years of age [2]. The most frequent (46.4/1000) genetic diseases were accounted for by multi-factorial diseases present at birth or before age 25 years. Single-gene disorders, present in 3.6/1000, included autosomal-dominant (1.4/1000), autosomal-recessive (1.7/1000), and X-linked recessive disorders (0.5/1000), followed by chromosomal anomalies in 1.8/1000, and genetic diseases with unknown etiology in 1.2/1000 [2]. These authors excluded all congenital anomalies that used to be included in estimates of genetic diseases before their study [2].

Almost 37 years after the analysis by Baird et al., as of 20 December 2024, the Online Mendelian Inheritance in Man^®^ (OMIM^®^) comprehensive compendium of genetic diseases [3,4] included a total of 27,626 entries comprising 26,107 autosomal, 1383 X-linked, 63 Y-linked, and 73 mitochondrial genetic diseases [5]. In OMIM^®^, the gene was described for 63% (17,449/27,626), and the molecular basis was known with a phenotype description for 23% (6391/27,626) of all described genetic diseases [5]. At the same time, on 22 December 2024, 11,074 clinical diseases, including 8199 disease—gene relationships and 114,961 phenotypic annotations, were reported on the website by Orphanet, a publicly available epidemiologic database based on worldwide or European estimations [6]. Orphanet tabulated 4363 individuals or families with rare diseases with incidence or prevalence information as of October 2024 [7]. At the population level, approximately 10% of all individuals are estimated to be affected by rare diseases [8]. Of note, the rarity of a disease depends on the context of variables such as geographical location and ethnic origin, with some ethnicities having a higher prevalence of certain diseases [8]. Geographically, in the USA, a disease is defined as rare if it occurs in less than 200,000 individuals. In contrast, the definition of a rare disease in the European Union since 2000 is based upon its prevalence being less than 1 in 2000 people [8]. The average time to obtain an accurate diagnosis for a rare disease is over six years, with avoidable costs of delayed diagnoses exceeding USD 500,000 per patient [9]. Genetic diseases comprise approximately 72% of the reported rare diseases, of which 70% are exclusively pediatric in onset [10].

More than three out of ten children with a rare disease die before their fifth birthday [11]. About 28% of deaths in the neonatal intensive care unit (NICU) are due to underlying genetic diseases [9], with 22% [12,13], 33% [14], and 41% [15] of all infant deaths reported as being due to underlying genetic diseases in U.S. cohorts [12,13,14,15]. In a single NICU in China, among 223 deceased newborns with 13 days as the median age at death, genetic findings were identified after death by genomic sequencing in 19.7% (44/223), including medically actionable disorders in 31% (13/42) based on genetic diagnosis [16].

A retrospective study of 1444 infants in a level 4 NICU in the Netherlands showed that 32% had a congenital abnormality [17]. Until the age of 2 years, 13% (194/1444) were genetically tested, with 37% (72/194) diagnosed, including in children without a congenital abnormality. Insufficient testing led to several genetically undiagnosed patients, highlighting the need for rapid and effective testing in NICUs [17].

Furthermore, it has been shown that ill infants with a genetic condition stay longer in the NICU than infants without a genetic condition. In a study published in 2019, the increased NICU stay costs for infants with a genetic diagnosis were USD 246,610 per patient when averaged for 117 patients. The total charges were greater than an additional USD 28,000,000 for all infants with a genetic condition in a single-level 4 NICU over 2 years at a tertiary care medical center in the USA, compared with infants without a genetic diagnosis [18]. The high incidence of genetic diseases in the neonatal period, which are often not easily recognized clinically due to incompletely developed disease phenotypes in infants, and the high costs of NICU stay, which could be avoided or decreased by rapid, effective testing, add to the need for a change in the way genetic testing is currently approached in critically ill infants and children.

Historically, cytogenetic karyotypic analysis by chromosomal banding techniques has been the primary tool used to examine the whole genome for genetic abnormalities since the late 1950s, when the correct number of chromosomes was ascertained to be 46, followed by the use of chromosomal microarrays (CMAs) since the 1990s [19,20]. The process of mapping genes to specific genetic loci on the chromosomes began in 1968, with rapid advances in the next two decades, as described by McKusick [21], which subsequently led to the initiation of the Human Genome Project [22,23].

When the Human Genome Sequencing project was completed two years ahead of schedule in April 2003 [24], about 92% of the nucleotide bases comprising the entire DNA sequence of the human genome were determined. The remaining 8% of the genome consisted of highly repetitive DNA sequences that could not be sequenced by the techniques available in 2003. That major landmark in 2003 formed the foundation for significant strides that occurred subsequently to unravel the genetic and genomic basis of human diseases, beginning with inherited genetic diseases and cancer. In 2010, CMA was a first-tier clinical diagnostic test compared to conventional cytogenetics for individuals with developmental disabilities and congenital anomalies [25]. Simultaneously, after the human genome was sequenced, massively parallel sequencing, or next-generation sequencing (NGS) techniques, including whole-exome sequencing (WES) and whole-genome sequencing (WGS), were applied to sequence the human genome. Comparing the human genome, in part or whole, in the individual with the disease, to the reference human genome then started to provide an understanding of the errors in the genetic code that led to various genetic diseases.

The currently used diagnostic tests for suspected genetic diseases include CMA and NGS using targeted gene panels, each with distinct capabilities and limitations. A CMA detects submicroscopic chromosomal losses and gains and copy number variants (CNVs) and does not require dividing cells, unlike karyotypic or chromosome banding analysis. However, a CMA cannot detect the following abnormalities: (1) balanced rearrangements, (2) single-nucleotide changes, (3) low-level mosaicism, i.e., the presence of abnormalities such as CNVs in only a few cells, and (4) copy number changes below the resolution of the microarray [26,27]. In contrast, targeted gene panel NGS identifies DNA sequence variants, including single-nucleotide variants, insertions, and deletions, but not CNVs or structural rearrangements. Targeted gene panels consist only of exons or regions of genes known to cause the specific diseases being tested for; therefore, targeted gene panels cannot identify abnormalities in any novel gene not included in the panel [27,28,29].

Furthermore, NGS, using WES, sequences all exons from protein-coding genes and exon–intron junctions and requires exon-capture and polymerase chain reaction (PCR) amplification steps during the pre-sequencing laboratory procedure. In contrast, WGS interrogates the entire genomic DNA sequence, including all exons, introns, and the deep intronic and regulatory gene regions, which can harbor causative abnormalities undetectable by WES. Since WGS analyzes the entire genomic DNA, its pre-sequencing laboratory procedure does not require exon capture and PCR amplification. Therefore, WGS leads to more uniform coverage of the regions analyzable by WES than achieved by WES, including in GC-rich first exons, and allows greater sensitivity in detecting CNVs [30,31,32], as also shown in clinical diagnostic settings [33,34]. Even if a CMA and WES are combined for diagnostic testing, variants such as balanced chromosomal translocations, small CNVs (<2 exons), and trinucleotide repeat disorders are missed [34]. In addition, deep intronic variants, copy-neutral inversions, complex structural rearrangements, and tandem repeat expansions are only identifiable by WGS, not WES [33,34,35].

The sequencing of the remaining 8% of the genome was completed using long-read technology and reported in April 2022 [36,37]. Meanwhile, with significant advances since the completion of the Human Genome Project, WGS has become a first-tier test in diagnosing critically ill infants with a genetic disease. If deemed clinically necessary in critically ill infants and children, WGS can be performed rapidly with a genomic molecular diagnosis in less than 24 h [38]. This review is meant for students and clinical practitioners who may not be experts in genomic sequencing and, therefore, is prefaced by a brief historical introduction. This work aims to chronologically review significant clinical studies worldwide in pediatric genomic sequencing that have led to the development of rapid WGS (rWGS) as a first-tier genetic test in the diagnosis and clinical management of critically ill infants and children with suspected and undiagnosed genetic diseases. These studies are followed by discussing the factors that will help to develop rWGS as a clinical test in critically ill infants, including evaluation of the clinical utility, accessibility to genetic testing, education for parents and providers, cost-effectiveness, ethical challenges, consent issues, secondary findings, data privacy concerns, false-positive and false-negative results, challenges in variant interpretation, costs and reimbursement, the limited availability of genetic counselors, and the development of evidence-based guidelines, which would all need to be addressed to facilitate the implementation of pediatric genomic sequencing in an effective widespread manner in the era of precision medicine.

## 2. Earliest Studies of Clinical Whole-Exome and Whole-Genome Sequencing in Genetic Diseases, Until 2012

Table 1 chronologically lists and summarizes the earliest clinical studies of WES and WGS in individual patients or families, including their significance [39,40,41,42,43,44,45,46,47,48,49]. These studies paved the way for developing clinical exome and genome sequencing studies in genetic diseases.

The exome comprises ~1% of the entire genome [39] and includes the protein-coding regions of ~22,000 genes scattered across the genome. In 2009, Ng et al. captured the gene targets in the exome, followed by massively parallel sequencing of the exome in eight previously characterized healthy individuals [50] and four unrelated individuals affected by a rare genetic disease [39]. The latter is an autosomal-dominant disease with severe multiple congenital contractures (Freeman-Sheldon syndrome, OMIM #193700) caused by mutations in the embryonic myosin heavy chain gene, *MYH3*, which had been determined earlier as the cause of this syndrome [51]. That WES study with four individuals of Yoruba Nigerian ethnicity, six European Americans, and two Asians showed that exome sequencing could identify disease-causing allelic variants in genetic diseases [39]. Next, the same group of investigators studied four individuals (two siblings and two unrelated) with Miller syndrome [41], for which the cause of the disease was unknown at that time. In these individuals with Miller syndrome, WES identified the molecular genetic basis of the autosomal recessive inherited disease, i.e., disease-causing variants in the gene encoding for a key enzyme in the pyrimidine de novo biosynthesis pathway, dihydroorotate dehydrogenase, *DHODH* [41]. Subsequently, also in 2010, Hoischen et al. used WES to show the de novo nature of the heterozygous mutations in SET binding protein 1, *SETBP1*, in the dominantly inherited Schinzel-Giedion syndrome [43,52]. From 2009 to late 2012, there were more than 300 publications for gene discovery by WES in genetic diseases, of which recessively inherited conditions comprised >115 novel genes in a total of >180 novel genes discovered in genetic diseases, excluding cancer [53].

The capability of WGS to reveal the molecular basis in genetically heterogeneous diseases was first demonstrated by Lupski et al. in 2010 when they performed WGS in the proband in a clinically diagnosed family with an autosomal recessive Charcot-Marie-Tooth disease Type 1 [42]. Before their study, seven human genomes had been characterized, including those of two individuals with the surnames Venter [54] and Watson [55] in 2007 and 2008, respectively. Five HapMap samples were also studied: one Chinese, two African (same HapMap sample twice-sequenced with different coverage), and two Korean individuals [42]. Those eight human genomes showed a total of 3.425 × 10^6^ (median; range 3.07–3.86 × 10^6^) single-nucleotide polymorphisms per genome [42]. Concurrently, in 2010, Sobreira et al. applied WGS in the proband in a family with metachondromatosis (OMIM #156250), an autosomal-dominant disorder [44]. They identified the molecular causes in two families with the same disease as loss-of-function mutations in exon four of the *PTPN11* gene [44].

In 2011, Worthey et al. performed WES in a 15-month-old child with a Crohn’s-like inflammatory bowel disease that was non-responsive to treatment. The diagnosis by WES, unsuspected clinically, led to a complete resolution of the colitis after a hematopoietic stem cell transplant [46]. Also, in 2011, WGS was used to identify the molecular basis of disease in 14-year-old fraternal twins who had been clinically diagnosed at the age of five years with dopa-responsive dystonia, enabling additional therapy that led to clinical improvement in the symptoms [47].

In 2012, WES was applied to show that de novo mutations cause unexplained intellectual disability [48]. Also, in 2012, less than a decade after the Human Genome Project was completed in 2003, Dr. Kingsmore’s group at the Children’s Mercy Hospital in Missouri, USA, performed rWGS in 50 h as a proof-of-concept in a research setting for acutely ill infants [49].

## 3. Key Cohort Studies, Whole-Exome and Whole-Genome Sequencing, 2013–2017

Table 2 chronologically lists key cohort studies published during 2013–2017 for WES and WGS performed in patients and families [56,57,58,59,60,61,62,63,64,65,66,67,68,69,70,71,72,73,74,75,76,77,78,79]. This list does not include all studies published during this time and should not be construed as such. Key studies that included large numbers of patients and had clinical impact are included in this table, along with a few smaller cohorts with 15–25 acutely ill infants. The diagnostic yield was not a consideration when including studies in this table. The table includes the month and year of publication, the institution and country where the studies were performed, study and cohort characteristics, genomic sequencing molecular diagnosis rates, and cost savings, if reported. A separate column indicates whether infants in NICUs were included in the study since the results for acutely ill inpatients in NICUs versus ambulatory children would have economic implications. The criteria for diagnoses in these studies were based on the five-tiered American College of Medical Genetics and Genomics (ACMG) guidelines for variant interpretation, with the pathogenic/likely pathogenic classification of identified variants required for positive molecular diagnoses [80].

In the U.S.A., the earliest clinical WES findings were published in late 2013 from a single institution [56]. That study was followed by retrospective reports of clinical WES from additional U.S. institutions and clinical reference laboratories in 2014 [56,57,58,59,60], 2015 [64,65], and 2016 [69]. The diagnostic yield of WES in these cohorts varied from 25% to 41% [56,57,58,59,60,64,65,69]. The diagnostic rates of WES (~30%) were higher than 5–15% by karyotype analysis and 15–20% by CMA analysis [65].

The first study to demonstrate the effectiveness of rWGS based on the acuity of illness in acutely ill infants and children in intensive care units (ICUs) was published in 2014, with a diagnosis rate of 73% by rWGS within 50 h [61]. The same group of investigators then retrospectively showed that 57% of acutely ill infants under 4 months of age were diagnosed by rWGS (“STATseq”) in comparison with 9% by standard genetic tests [63]. Those STATseq reports were delayed with a median time to report of 23 days for several reasons, including the presence of several new or developmental components of the WGS process at the time of study, such as software development for structural variant detection, novel disease-gene phenotypes, or variant phenotypes that required extensive analysis and external expert consultation [63]. The next version of rWGS was developed and completed in 26 h to apply in conditions in the NICU where rapid diagnosis could prevent significant morbidity and mortality [66].

Outside the U.S.A., in 2015, clinical WES results were reported from the U.K. for their Deciphering Developmental Disorders national study [62] and the 500 human genomes project by WGS [33]. Similarly, in 2016, results of Canadian sequencing studies were reported for clinical WES in their national rare disease genes project, a pilot next-generation sequencing (NGS) study in newborns and infants with a panel of 4813 disease-relevant genes [70], and by WGS in 100 pediatric patients [67].

In Australia, Stark et al. prospectively reported a more significant (57.5%) diagnostic yield of singleton (proband only) WES as a first-tier test from patients evaluated during 2014–2015, compared with 13.75% by standard tests. In their study, the cost of care with WES as a first-tier test was much lower than standard care (numbers given in Table 2) [75]. Similar results were reported in 2017 by the Melbourne Genomics Health Alliance, which had a 52% diagnosis rate, including cost savings by WES compared to the standard genetic pathway [77].

In 2017, results followed from a German study of 1000 globally situated patients who underwent WES [72] and a Dutch study of non-acute pediatric patients [76]. The WES diagnostic rate in both studies was close to 30% [72,76]. In late 2017, rapid genomic sequencing in critically ill infants was reported by rapid singleton WGS in a Dutch study [78] and by critical trio (proband and both parents) rapid WES (rWES) in a study from Texas, USA [79]. In the latter study by Meng et al., 50.8% of critically ill infants were diagnosed by the rapid trio WES with a median turnaround time (TAT) of 13 days, with a change in management in 72% of those rapidly diagnosed critically ill infants [79]. As summarized in Table 2, these results were significantly better than proband-only or regular trio WES, indicating the critical importance of TAT in diagnostic genomic sequencing for critically ill infants.

Interestingly, in a large U.S. study of WES and WGS, WES was replaced by WGS during the study [73]. Re-analysis of WES/WGS data improved the diagnosis rate [73], as also observed in another WGS study from the USA at the same time [74] and in the 2015 national U.K. study with re-analysis reported in 2018 [81].

In the studies in Table 2, the diagnosis rate corresponded with identifying pathogenic/likely pathogenic variants as classified by widely accepted ACMG criteria [80]. Regarding secondary (earlier termed incidental) findings, which are found in genes not related to the primary diagnosis, the ACMG guidelines recommend that in clinical genomic sequencing studies, deleterious variants be searched for in the list of genes for secondary findings irrespective of the primary clinical phenotype [82,83]. In contrast, the Canadian guidelines do not support the intentional searching of variants in genes unrelated to the primary indication for genomic sequencing, but if such a variant might be inadvertently identified, adults are given the option to receive those results; in children, incidental findings that are highly penetrant and actionable in childhood must be returned [84]. Therefore, Daoud et al. conducted their study with NGS with a panel of 4813 disease-relevant genes [70]. The European guidelines also do not advocate actively searching for variants in genes unrelated to the primary indication for sequencing [85]. Notably, to call a variant causal in the WGS study from the Wellcome Trust Center for Human Genetics required additional evidence such as functional data, familial transmission, de novo status, and screening of other patients [33]. Those observations also apply to variants detected in clinical cases examined by WES, as exemplified in a prior case report [86].

The reader is referred to a previous article for additional genomic sequencing case reports and clinical WES studies published from January 2009 to November 2017 [87].

## 4. Key Cohort Studies, 2018 to 2024, for the Development of Rapid Whole-Genome Sequencing (rWGS) in Pediatric Patients

Table 3 chronologically summarizes 35 major sequencing studies performed in twelve countries, including the USA, Canada, UK, Australia, Sweden, China, Poland, France, Belgium, the Netherlands, Israel, and Brazil, from 2018 to 2024 [88,89,90,91,92,93,94,95,96,97,98,99,100,101,102,103,104,105,106,107,108,109,110,111,112,113,114,115,116,117,118,119,120,121,122,123,124,125,126,127,128]. Like Table 2, this list does not include all studies published during this time and should not be construed as such. Key studies that included large numbers of patients and had clinical impact are included in this table, along with a few smaller cohorts with 15–25 acutely ill infants. In addition, an rWGS study performed in the Netherlands is summarized in Table 2 [78]. Table 3 includes the month and year of publication, the institution and country where the study was performed, the study and cohort characteristics, the genomic sequencing molecular diagnostic yield, and additional significant inferences, including the TAT, cost savings, and parental and clinician perceptions, if reported. Similar to Table 2, a separate column indicates whether infants in NICUs were included in the study. Like Table 2, a positive diagnosis in these studies required pathogenic/likely pathogenic classification of identified variants [80].

The “Newborn Sequencing In Genomic Medicine and Public Health-1” (NSIGHT1, ClinicalTrials.gov accession NCT02225522) trial in the USA hypothesized that rWGS would improve the rate of genetic diagnoses in the NICU and PICU within 28 days [88]. The TAT for rWGS was extended by 7 to 10 days because the trial protocol required confirmation through orthogonal methods, with all rWGS findings confirmed by Sanger sequencing. Additionally, the trial required clinicians to suspect a genetic disorder, which often necessitated a standard test before enrollment, and informed consent was needed from both parents. All these factors affected the time to diagnosis by rWGS, resulting in a time longer than the expected median time of 5 days. Despite these delays, rWGS diagnosed 31% (10/32) of infants, an increase of 28% (*p* = 0.003) compared to the 3% (1/33) of infants diagnosed by standard tests alone. The trial compared rWGS with standard tests but was terminated early, after 21 months, as targeted NGS, WES, and WGS became available as standard tests [88].

The following study from the same institution demonstrated that rWGS has clinical utility, enhances clinical management, and reduces healthcare costs; therefore, it should be regarded as a first-tier test for acutely ill infants with suspected genetic etiology [89]. Concurrently, a meta-analysis of 37 studies on WES, WGS, and CMA, which included over 20,000 children, showed that pooled WES and WGS studies had superior diagnostic utility compared to CMA, suggesting that WES or WGS should be the first-tier genetic tests [91]. Subsequently, Clark et al. expedited rWGS to a median of 20 h and 10 min for genetic diagnosis by employing automated clinical natural language processing (CNLP) to extract phenotypic features and facilitate diagnosis [95]. The CNLP-extracted phenotypic features corresponded to the diseases identified in the 101 rWGS-diagnosed children. Notably, CNLP identified 27 times more features than expert manual interpretation. This reduction in reporting time meant that, among subsets of patients, early diagnosis by rWGS could prevent morbidity or mortality, with examples including neonatal epileptic encephalopathies, metabolic disorders, and septic shock potentially due to immunodeficiency. The prospective use of their rapid platform correctly diagnosed 3 of 7 seriously ill NICU infants, resulting in time savings, and in each case, the diagnosis influenced treatment decisions [95].

At the same time, in 2018, WGS and WES were compared in pediatric patients in Canada, with WGS diagnosis in patients including variants not detectable by WES [90]. In Australia, rWES eliminated the cost of increased ICU days, additional tests, and subspecialty visits after confirmatory diagnosis [93]. Rapid WGS clinical analysis in the U.K. with a median TAT of 8 days showed a 42% diagnostic rate in the pediatric ICU (PICU) [92], while a standard WGS study with broad inclusion criteria showed an overall 21% diagnosis rate, with higher diagnosis rates in specific phenotypes in the NICU and PICU [94].

The NSIGHT2 randomized clinical trial in the USA subsequently evaluated the effectiveness and outcomes of two rapid genomic sequencing methods (rWGS and rWES) along with two analytic approaches, singleton proband, and family trio, in seriously ill infants with unknown genetic etiologies [96]. The effectiveness of the diagnosis was assessed based on the diagnosis rates, time to diagnosis, clinical utility, perceived family utility, and cost [96]. This trial was notable for several reasons:

(1) 98% of seriously ill infants under 4 months of age (*n* = 1248) were screened for eligibility, encompassing a much larger group of infants, including those with a lower probability of genetic diseases than in previous studies. This characteristic resulted in a less biased estimate of the incidence of genetic diseases in seriously ill infants, determined to be at least 14% in the regional intensive care units in San Diego.
(2) Enrollment in the trial occurred within 96 h of admission or the onset of abnormal symptoms, indicating that rWGS was evaluated as a first-tier test without prior specialist consultations. This restriction excluded 365 eligible infants, including 24 (7%) who received rWGS outside the trial, with 4 (17%) diagnosed with rWGS.
(3) Ultra-rapid WGS, yielding a median time to result of about 2.3 days compared to 11.3 days for rWES or rWGS, was not fully technologically developed and had to be conducted more expensively for most of the trial enrollment period. Consequently, only the most seriously ill infants received ultra-rapid WGS.

As anticipated, the NSIGHT2 trial showed that the analytical performance of rWGS was superior to that of rWES, identifying twice as many pathogenic and likely pathogenic nucleotide variants. However, the diagnostic yield was similar for rWGS (19%, 18/94) and rWES (20%, 19/95). This difference between the enhanced analytical capabilities of WGS and the diagnostic yield likely stemmed from the fact that coding variants remained the primary basis for diagnoses. With an increasing understanding of non-coding variants and their role in disease pathogenesis, WGS is expected to yield more diagnostic results than WES. Notably, 98% (50/51) of diagnoses were achieved through singleton sequencing, promoting the presumptive identification of de novo, typically novel variants. Trio sequencing, used in the study to validate the phasing and de novo variants identified by singleton sequencing, resulted in a diagnosis for only one infant. The cost of trio sequencing was twice that of singleton analysis. Nonetheless, the TAT for the singleton analysis was extended by approximately five days due to the additional time required to confirm the findings from the singleton analysis [96].

Indeed, similar to the increased analytic capability of WGS in the NSIGHT2 trial, the validation and development of the WGS pipeline in comparison with CMA in the genetics clinics in Stockholm, Sweden, confirmed that WGS detected all known structural variants that had been detected by CMA in patients with intellectual disability [34]. WGS identified additional variants and clarified the precise nature of chromosomal rearrangements and complex abnormalities [34]. In their efforts toward large-scale national collaboration, the same genomics group in Sweden demonstrated an overall 40% diagnosis rate by WGS among their 3219 patients, with the diagnosis rates higher among specific disease groups [107]. As examined during 2015–2019, the rates of WGS diagnosis included 54% in skeletal dysplasia, 50% in hydrops fetalis, 42% in neuromuscular and ataxia disease, 41% by their OMIM morbid gene panel with 3959 genes, 39% in intellectual disability and malformations, 34% in pediatric hepatology, 32% in inherited metabolic including mitochondrial diseases, 31% in severe infantile epilepsy, 29% in immunologic diseases including neutropenia, 28% in connective tissue diseases, 20% in inherited cancer, and 19% in neurodegenerative disorders [107]. Notably, all these diagnosis rates were achieved by singleton analysis, except for infantile epilepsy and the OMIM morbid gene panel, for which trio samples were analyzed [107].

For acutely ill infants with congenital heart disease, rWGS showed a high diagnostic rate of 46% compared to just 10% from standard clinical tests, which included panel NGS and CMA, in a retrospective analysis of a U.S. cohort [108]. Evaluation by rWGS led to changes in management regardless of whether a positive diagnosis was obtained. Given that congenital heart disease is a common congenital anomaly that appears early in the neonatal period and incurs high hospitalization costs (with an average total hospitalization cost exceeding USD 900,000 compared to the approximate cost of rWGS at USD 8500), the study recommended that rWGS be used early in this specific group of acutely ill infants [108].

Other countries where rapid or ultra-rapid genomic sequencing has been utilized in pediatric genetics include (1) China, with an overall diagnosis rate of 69.6% via rWES [100], (2) Poland, with a diagnosis rate of 72.2% via rWES [104], (3) Australia, with an overall diagnosis rate of 51% through ultra-rapid WES, the highest being 56% in the NICU compared to 47% in the PICU [101], (4) France, with a 49% diagnosis rate [118], (5) Belgium, with a 57.5% diagnostic rate [121], and (6) Israel, with a 50% diagnostic rate [122]. Focusing on PICU patients, a similar diagnosis rate of 45% was noted in the PICU in the retrospective rWGS study by Sanford and colleagues, where 74% of patients had been nominated for rWGS by intensivists [97], and in the prospective U.K. study in which subspecialty consultants nominated PICU patients for rWGS [92].

The prospective multi-year Genomic Medicine in Ill Neonates and Infants (GEMINI) trial in the USA compared targeted sequencing of 1722 actionable genes, performed at a large clinical reference laboratory, with rWGS, performed at Rady Children’s Hospital [109,110]. The final results of this trial, reported in 2023, are shown in Table 3. Secondary findings were not actively sought to be identified in the GEMINI trial, but using the human phenotype ontogeny framework for the clinical phenotype [129] to help with analyzing the variants led to their identification [110]. Notably, an interim analysis of 133 infants in the GEMINI trial in 2021 revealed challenges that must be addressed for WGS to become a first-tier test [109]. There was only a 47% inter-laboratory concordance in the results for diagnostic variants, with even lower concordance (14%) for variants of uncertain significance (VUS), while negative results showed 100% concordance [109]. One enrolled infant received two diagnoses, each identified by one of the two laboratories. Even the interim findings in the GEMINI trial underscored previously recognized inter-laboratory differences in the interpretation and classification of variants, where the overall five-category concordance rate for germline variants among nine laboratories was only 54% [130].

Another inter-laboratory study revealed that clinically significant diagnostic germline variants (both pathogenic and likely pathogenic) identified through panel-based NGS were reported inconsistently by seven laboratories across various clinical genetics contexts, including pediatric genetics and newborn disorders [131]. These clinically relevant variants—such as large indels, complex rearrangements, small copy number variants, low complexity repeat-associated variants, segmental duplications, pseudogene associations, or putative mosaicism—were collectively common, even though they were individually rare within the larger cohort. The technical challenges associated with detecting and classifying these variants increased the likelihood of false negative results for one in seven clinically relevant variants. Notably, the findings from this study are equally applicable to WES and WGS used in any clinical setting, including pediatric genome sequencing [131].

Non-ACMG-recommended incidental genetic findings were the focus of a 2022 publication, which described 23 such incidental findings in 21 pediatric patients in four studies, including the SouthSeq study, who had undergone genomic sequencing [132]. Incidental genetic findings are medically relevant but unrelated to the indication for testing, and their identification is not sought during testing [132]. In contrast, secondary findings are related to the indication for testing and are specifically sought to be identified [83,132]. Importantly, acting on incidental findings can be beneficial or harmful for patients, and a plan must be in place for which incidental findings will be disclosed to participants and how [132,133,134].

### 4.1. A Comparison of Targeted Panel Sequencing, WES, and Rapid WGS (rWGS) in Critically Ill Children with Suspected Genetic Diseases

Table 4 compares the interrogated genomic regions, TAT, diagnostic yield, cost, and clinical utility of rWGS with WES and targeted panel NGS in pediatric genomic sequencing.

### 4.2. Trio WES and Trio rWGS May Have an Increased Diagnostic Yield, Decreased Turnaround Time (TAT), and Increased Cost Compared with Proband-Only WES and Proband-Only rWGS

Trio WES and trio WGS include sequencing both parents along with the proband, which helps to identify de novo disease-causing variants in the proband, which will be absent in the parents. Genomic sequencing by WES and WGS leads to many variants flagged in the clinical workflow and requires interpretation of their clinical significance. Having the parental sequences for comparison with the proband’s sequence helps to reduce the number of variants that would be flagged, as was shown in a 2015 WES publication [62]. Filtering the variants based on familial analysis allows for a more thorough analysis of the flagged variants and reduces the possibility of missing a de novo variant [64]. That is the most likely explanation for why the diagnosis rates by trio WES and trio WGS have been observed to be higher than proband-only WES and WGS [59,62,64,79].

The number of flagged variants in WGS is much higher than in WES. That number is reduced by trio WGS compared to proband-only WGS, shortening the time required to analyze the flagged variants and render a diagnosis. Therefore, in urgent clinical situations where ultra-rWGS was deemed necessary, trio ultra-rWGS was performed in Project Baby Bear [101]. Since trio WGS requires processing three samples, its costs are higher than singleton WGS, which requires only one sample to be processed. Therefore, an option to use Sanger sequencing analysis of parental samples was used in conjunction with proband-only WGS in the 2022 study by Bowling et al. [114]. As discussed above, however, high diagnostic rates were achieved with singleton WGS in the NSIGHT2 trial in the USA [96] and in studies of WGS from Sweden [107].

### 4.3. Why Can the Diagnostic Results of Genomic Sequencing Vary Between Different Laboratories?

The diagnostic results between different laboratories may vary depending primarily on two broad reasons: (1) differences in the assays developed and validated for use in the clinical laboratory, including the design, purpose of the clinical assay, the targets included, and metrics established during assay validation. Thus, providers ordering these tests should always understand the clinical assays before ordering to confirm that the findings they are looking for would be identified by the specific test from any laboratory. (2) Secondly, laboratories may differ in the clinical significance assigned to some detected variants. Interpretation of variants was the primary reason stated to be the cause of diagnostic discrepancies in the GEMINI trial of rWGS in the USA [109,110] and in another study, as discussed above [131].

### 4.4. Assessment of the Clinical Utility of WES and WGS in Critically Ill Infants and Children with Suspected Genetic Diseases

The clinical utility of WES and WGS studies has been defined heterogeneously across various studies. A precise clinical diagnosis holds great value, even though achieving such a diagnosis may not always result in better treatment outcomes or prevention at the time of diagnosis [135]. As previously noted, the benefits extend to individuals, families, and society [135]. In a meta-analysis published in 2019, the authors identified the superior clinical utility of WGS as a first-tier test over CMA [91], which is currently recommended as a first-tier genetic test [25,136]. While both WES and WGS studies demonstrate clinical utility, no significant difference was observed between the two methods in the meta-analysis [91].

In the NSIGHT1 clinical trial, actual clinical utility was evaluated based on the changes in management after molecular diagnosis by WGS and not on the potential to change management with any given diagnosis. This evaluation was based on chart reviews and surveys with referring clinicians, who were themselves learning about the diagnoses revealed by WGS, thus limiting the utility of this first NSIGHT1 trial [88].

In the subsequent NSIGHT2 trial, the treating physicians for 201 (94%) of 213 enrolled infants completed surveys designed to evaluate clinical utility, changes in management, and any perceived harm due to genomic sequencing. In 154 (77%) infants, diagnostic sequencing was perceived to be “useful” or “very useful,” notably including 72% (112/156) of infants with negative results by genomic sequencing. The utility was noted to be greater (93%, *n* = 42/45; *p* = 0.0023) with positive results [96]. Clinical management changed in 28% (*n* = 57) of infants, with significantly higher rates with positive genomic sequencing results (63%, *n* = 31/49) and ultra-rapid WGS (63%, *n* = 15/24). In comparison, fewer infants (23%, *n* = 42/183) examined by rWES or rWGS had a change in management [96]. Similarly, improved communication between families was noted by clinicians in 41% (84/207) infants, with more improvement noted with positive genomic sequencing results (69%, *n* = 34/49) and ultra-rapid WGS (67%, *n* = 16/24) than with rWES or rWGS (37%, *n* = 68/183) [105].

In the NSIGHT2 trial, the parents of 83% (*n* = 176/213) and 55% (*n =* 117/213) of infants completed enrollment and one-week post-result surveys, respectively [106]. Interestingly, 97% of parents found the genomic sequencing tests “useful” or “somewhat useful,” while only 23% (*n* = 27/117) of the corresponding infants received a positive diagnosis. Most families reported benefits for both the infant and the family. Only 3% of families indicated perceived harm in the surveys due to negative results; however, upon follow-up, these families expressed that they would make the same decision again regarding genomic sequencing [106].

In the Australian Genomics Health Alliance ultra-rapid WES study, 44% (*n =* 48/108) of patients experienced a change in management. This change rate was higher among the 55 patients who received a molecular diagnosis (76%, *n* = 42) compared to 11% (*n* = 6) among the 53 patients with a negative result [101]. In the latter case, the changes included discontinuing medications or canceling planned tests. The referring clinical geneticists found the positive reports, i.e., those with a diagnosis, to be “very useful” or “useful” for 95% (*n* = 52/55) of patients and neutral for 5% (*n* = 3/55). Conversely, the “negative” reports, i.e., without a diagnosis, were seen as “very useful” or “useful” for 58% (*n =* 31/53), neutral for 38% (*n* = 20/53), and “not useful” for 4% (*n* = 2/53) of patients [101].

In the U.K. study of trio rWGS, 42% (*n* = 10/24) received a molecular diagnosis, which resulted in an immediate change in clinical management for 3 (30%) individuals, in addition to disease-based management for all families with accurate genetic counseling [92].

The SouthSeq study, a Clinical Sequence Evidence-Generating Research (CSER) Consortium project, implemented WGS in acutely ill infants at various clinical sites across the Southeastern USA and included a majority of racially and ethnically diverse populations that have historically been underserved in genomic medicine [114]. In addition to the WGS diagnosis and related findings presented in Table 3, which demonstrated the clinical utility of WGS over standard genetic testing, the authors discovered that infants with specific phenotypes were more likely to receive a positive diagnosis, defined as definitive or likely diagnoses, through WGS. Among 13 grouped phenotypes for all infants in the study, the highest likelihood of a positive diagnosis was observed in infants with abnormal craniofacial, ophthalmologic, auditory, skin, or hair findings (*p* < 0.0001), followed by gastrointestinal phenotypes and neurological/muscular features [114].

A review of 33 clinical studies performed from 2012 to November 2021, which included 15 rWGS, 18 rapid WES, and one rapid panel test, revealed a weighted average diagnostic rate of 36% (ranging from 19% to 83%) among 2874 critically ill children in ICUs [137]. In that review, the weighted average rate of change in management following the return of results was 27% (ranging from 7% to 60%) [137]. A subsequent review published in February 2024 of 44 studies of ultra-rapid WGS, rWGS, or rapid WES in 3609 infants and children showed a weighted average diagnostic rate of 37% (ranging from 19% to 83%) [138]. Thirty-nine of these 44 studies reported a change in management after the return of results, with a weighted average rate of 26% (ranging from 7% − 63%) [138].

A separate systematic review assessed the clinical utility of WES and WGS studies in critically ill infants and reported on 21 prospective studies conducted up to May 2022 [139]. This review included a total of 1654 infants admitted to any ICU; a positive genetic test was reported in 46% (with a range of 15% to 72%) of infants, and utility was recognized in a mean of 37% (ranging from 13% to 61%) [139]. That study indicated that the measurement of utility varied across studies, and changes in management were emphasized rather than meaningful benefits. Additionally, several important features were missing from most studies: patient-reported benefits in 95% (*n* = 20/21), the utility of negative or uncertain results in 71% (*n* = 15), and potential harms in 95% (*n* = 20) [139]. The editorial accompanying this systematic review emphasized that moving toward standardizing the utility of genomic medicine in critically ill infants is necessary for its effects to be realized at the individual patient and population levels [140].

Importantly, in the 21 systematically evaluated prospective studies until 2022, utility was reported to be higher in only small cohorts, with larger cohorts reporting significantly lower utility (*p* = 0.002) [139]. A Dutch study in 2023 defined the clinical utility of rapid WES in critically ill infants based on increased diagnostic yield, shorter time to diagnosis, and net healthcare savings [122]. In 2024, a study from Israel showed a high diagnostic rate in 130 infants, as shown in Table 3, possibly due to two reasons: (1) chromosomal abnormalities were included in the diagnostic yield, and (2) there were consanguineous families in the cohort with autosomal-recessive disorders identified by rWGS [124].

### 4.5. Cost-Effectiveness of Clinical WES and WGS

In the USA, among patients who underwent WES or WGS testing, the average cost of previous genetic tests was USD 19,100 per patient, according to a 2014 study on the effectiveness of WES and WGS in neurodevelopmental disorders [61]. Sequencing in the outpatient setting was determined to be cost-effective, with costs reaching up to USD 7640 per family or USD 2996 per individual [61]. In acutely ill infants admitted to the hospital, rWGS resulted in shorter inpatient stays and reduced professional fees, leading to estimated cost savings of USD 19,000 per infant sequenced by rWGS [89].

In Australia, in the prospective cost-effectiveness study of WES as a first-tier test that was published in 2017, the best scenario was the cost savings of AUD 2182 (USD 1702) per additional diagnosis achieved if singleton WES was used early in the diagnostic process to replace most of the standard tests [75]. For the other pathways, the average cost of standard care per diagnosis was AUD 27,050 (USD 21,099). If WES was performed after standard multiple genetic investigations, the cost increased per additional diagnosis by AUD 8112 (USD 6327). The cost of singleton WES was AUD 5047 (US 3937); if WES was used to replace some investigations, then that incremental cost decreased to AUD 2622 (US 2045) [75]. That study focused on children less than 2 years of age [75]. Similar cost savings for the early use of WES were prospectively reported for children aged 2–18 years by the Australian Genomics Alliance Group [77].

Interestingly, a systematic review in 2018 evaluated 36 publications of economic evaluations, costs, or outcomes of WES and WGS [141]. That review noted that the outcome measure in the included studies was the “diagnostic yield”, which is problematic for health technology assessment agencies such as the National Institute for Health and Care Excellence in the UK and the Pharmaceutical Benefits Advisory Committee in Australia. These agencies recommend life years or quality-adjusted life years (QALYs) as outcome measures since they allow interventions to be compared to decide the allocation of healthcare resources [141]. In that review, the health-economic evidence basis for using WES and WGS in clinical practice was described as very limited [141].

Based on clinical studies of pediatric genomic sequencing in acutely ill infants and children in the USA, rWGS was implemented clinically in five hospitals in California through Project Baby Bear for critically ill infants covered by the state’s health insurance plan [111]. Along with achieving a 40% diagnosis rate and a 32% change in management, rWGS resulted in significant savings from reduced hospital costs and professional fees [111]^,^ as outlined in Table 3. Subsequently, the state of Michigan approved insurance coverage for rWGS for admitted infants under one year of age [119,120]. Unlike a few states in the USA that have permitted insurance coverage for rWGS, the approval in Michigan enabled rWGS testing for infants in NICUs without placing the burden of genomic sequencing costs on the hospitals [142].

The cost-effectiveness of WES and WGS were estimated in 2022 in children aged <1 year and <18 years in the following situations: (1) standard of care (SOC) genetic testing, (2) WES, (3) WGS, (4) SOC followed by WES, (5) SOC followed by WGS, (6) WES followed by GWS, and (7) SOC followed by WES and subsequent WGS [143]. According to that study, first-line WGS may be considered the most cost-effective test in diagnosing critically ill infants with suspected genetic conditions [143]. WES is nearly as efficient an option when WGS is unavailable [143]. The authors assumed that infants being tested were severely disabled and children being tested were moderately disabled. Additionally, assumptions about lifetime trajectories were made with a broad prognostic spectrum from the least optimistic to the most hopeful. Subgroups of pediatric patients in whom WES or WGS would be most cost-effective are yet to be studied [143].

## 5. What Factors Would Need to Be Addressed to Facilitate the Adoption of WGS, a New Technology, into Care for Acutely Ill Neonates and Pediatric Critical Care?

Insights into this question were provided by interviewing multi-disciplinary healthcare professionals at the five medical centers for the state-wide implementation of rWGS in Project Baby Bear [144]. Qualitative analysis of those interviews led to an understanding of five common themes across all sites for implementing rWGS. These themes included:

(1) A Project Champion at each site who secured institutional support and then managed the project once that support was established.
(2) Education needs were considered vital, especially for physician providers, regarding rWGS implementation.
(3) The decision-making roles regarding ordering rWGS, reporting results, and following up necessitated negotiation and collaboration between the intensive care and genetics clinical services.
(4) Perceptions regarding rWGS included worries about the complexity of diagnostic information derived from rWGS, possible future negative impacts on patients or their families, and issues related to insurance coverage. Despite these concerns, all interviewees, including physicians, expressed optimism about the potential of rWGS in the critical care of newborns and pediatric patients.
(5) Clinical workflow processes for rWGS were illustrated to highlight the key areas with differences across sites [144].

Furthermore, Project Baby Deer showed similar positive responses to implementing rWGS across Michigan state children’s hospital sites [120,145,146]. The concerns related to education in genomics, potential negative future implications for patients or families, and insurance coverage were also similar [145].

As previously noted [147], the SouthSeq study identified the education of non-genetics providers as a critical determinant of the use of WGS in neonatal and pediatric critical care [115]. In the SouthSeq study, test cost, insurance coverage, and TAT for WGS were considered the most significant barriers to implementing WGS in the NICU [115].

In Australia, physicians strongly supported the implementation of WGS in an interdisciplinary care model [148]. The concerns included ethical issues, especially the possibility of finding unexpected secondary findings [148], which were previously discussed [149,150] and updated in 2021 with a list of 79 genes in the ACMG v3.0 update [151,152]. Five additional genes were added to the list of unexpected secondary findings in the ACMG v3.1. update in 2022 [153] and three additional genes in the ACMG v3.2 update in 2023 [154]. Post-WGS genetic counseling for secondary findings can be challenging, with examples discussed in an earlier publication [103]. Secondary findings are discussed further in the ethical challenges section of this paper.

### 5.1. Informed Consent for Rapid Genomic Sequencing in Infants and Children

Genomic sequencing for germline variants requires informed consent. In pediatric genetics, this involves explaining the diagnostic test procedure, potential benefits and risks, possible harms, the voluntary nature of the test, and alternative options available to the family to the parents or legal guardians of the child. This must be performed while respecting their autonomy and potential decision to refuse testing without consequences for clinical or social care, protecting their privacy and confidentiality, and clarifying how the generated data will be managed and shared in the future [155,156,157,158].

In an Australian study, the prioritization of ultra-rapid WGS results, the timing of ultra-rapid WGS, the adaptation of pre-test counseling, differences between clinical practitioner and family decisions, and prognostic uncertainty were described as challenging situations [159]. When rapid genomic sequencing is needed for an infant, the parents may still be adjusting to having a newborn in the family, and there may not be enough time for the parents to grasp all the nuances of informed consent [159]. Setting realistic expectations from rapid genomic sequencing, misalignment between healthcare practitioners and families, and the timing of the test causing problems with parent–child bonding have all been cited as challenges in pre-test counseling for rapid genome sequencing [159]. Divergent views have been reported among NICU practitioners regarding the specialty of the clinicians who should obtain informed consent for rWGS [160]. Some have insisted that genetics professionals should obtain consent even if there is a delay in proceeding with the test for a day and placing the patient at extra risk. In contrast, others prefer consent from any non-genetics professional to avoid delay. Nevertheless, there is agreement that whoever obtains the consent should be able to explain all relevant points to the parents and answer their questions [160].

It is unclear whether pre-test parental consent is necessary for rWGS in every case, as discussed in a study that examined the perspectives of English-speaking pediatric and neonatal intensivists primarily from Europe, Australasia, and North America [160]. The child’s best interests should determine the timing of testing, and guidelines for when testing could proceed without consent are needed. Additionally, the impact of genetic diagnosis on life-sustaining care needs further study [160].

The most common reasons cited when parents decline to participate in pediatric genomic sequencing are privacy and discrimination, lack of research interest, being overwhelmed by the current situation, concerns about psychological impact, and time commitment or study logistics [161]. Interestingly, only six families declined consent for rWGS in Project Baby Bear [111], which was offered as a clinical test. In contrast, between 35% and over 50% of eligible or approached families denied WGS for ill infants and children when the study was perceived as a research study, both in the USA during the NICUSeq [113] and NSIGHT2 [96] trials and in the French et al. study in the UK [94].

Even in healthy newborns offered newborn WGS in the BabySeq project [162], only 6.9% of the families approached consented to the study. Among the 1760 families who declined participation in the BabySeq project, research, including public sharing of research data, was the primary reason for declining [163].

These findings indicate that genomic sequencing must be offered as a clinical test, not on a research basis, for the procedure to benefit infants and children.

### 5.2. Ethical Challenges for Rapid Genome Sequencing in Critically Ill INFANTS and Children

The ethical challenges of genetic testing in children are amplified with genome sequencing due to the vast amounts of data generated by WGS [156]. Genomic sequencing leads to significant concerns regarding data privacy and the identification of unintended or secondary findings, which need to be disclosed to parents while obtaining informed consent. A 2021 systematic review of 143 publications from 3175 gives a detailed account of distinct ethical challenges identified in pediatric genomic sequencing [164]. These ethically challenging situations in pediatric genomic sequencing could vary in importance for each examined patient and family [164].

#### 5.2.1. Secondary Findings or Additional Findings

After the ACMG’s initial statement in 2013 [82], feedback from geneticists and ethicists led to an updated ACMG statement in 2015 that included the option to opt out of receiving secondary findings [165]. According to the 2021 review, ethical challenges surrounding the return of secondary findings are among the most frequently discussed topics in the literature [164].

Ethical dilemmas associated with secondary findings may arise when a potentially harmful additional finding, such as a cancer-predisposing pathogenic variant expected to manifest in adulthood, is identified in a child through genomic sequencing. Awareness of that genetic predisposition could benefit the parents and family, even if the child would not be affected by that knowledge until adulthood [164,166]. Who should decide which findings should be returned to the family? Should parental discretion determine what is in the best interests of the child? [167,168,169]. A Canadian study of parents who elected to receive secondary findings for an adult-onset condition through their child’s sequencing showed that a subset of parents wanted to know for the child’s sake but did not consent to testing themselves for that newly identified condition [170]. Among 23 parents from 18 families as participants, two factors were considered as positive for secondary findings: “They may enable prevention of/preparation for unanticipated health vulnerabilities in the child (17/23; 74%), and they may enable knowledge about and potential mitigation of parental health risks (12/23; 52%)” [170]. In contrast, “psychological distress (18/23; 78%), the potential for insurance discrimination (14/23; 61%), making sense of ambiguous findings (8/23; 35%), and managing the ’weight’ of inflicted insight (8/23; 35%)” were considered as the concerning aspects of secondary findings [170]. Additionally, ethical dilemmas may also arise if genomic sequencing reveals unexpected non-paternity, the disclosure of which by the providers to the family could lead to discord in the family that could potentially harm the child.

A systematic review indicated that the perspectives of providers and recipients of WES and WGS regarding secondary findings vary across a spectrum. The nuances in the situations of recipients who have received secondary findings should be considered when making decisions about these findings rather than relying solely on technical points [171]. Another systematic review indicated that the literature provided limited insights into how returning secondary findings has influenced precision health outcomes, emphasizing the need to prioritize rigorous research on recipients of secondary findings [172].

The key points regarding secondary findings are that the identified variants must be actionable, pathogenic variants for the disease, and their penetrance, medical implications, and clinical management should be discussed with the recipient [173]. However, the penetrance of all disease-causing variants remains unknown, with documented cases of individuals carrying pathogenic variants without clinical disease [174]. We also do not yet entirely understand the penetrance of disease-causing variants in healthy individuals. Therefore, conveying the risks, benefits, or potential harms of carrying a causal variant for a potentially life-threatening or life-altering disease to a healthy carrier who may or may not manifest the disease in their lifetime presents a significant ethical challenge, particularly in pediatric genomic sequencing, which could influence the perceptions of well-informed potential participants in genomic sequencing. In this light, the opinions of practitioners in the UK who returned these “additional findings” to recipients in their 100,000 Genomes project highlighted cautious optimism and the necessity of carefully handling these findings in pathways that would need to be developed to offer WGS clinically [175].

#### 5.2.2. Data Privacy Concerns

In the USA, the federal Health Insurance Portability and Accountability Act (HIPAA) Privacy Rule has been the primary foundation for protecting patients’ health information since 2003 [176]. The federal Genetic Information Nondiscrimination Act of 2008 (GINA) safeguards against discrimination in employment and health insurance coverage. However, GINA does not extend protection against life, disability, or long-term insurance [176]. Furthermore, the nature of genomic sequencing data is such that even de-identified data from genomic sequencing carries the risk of patient identification [177]. Determining how much of the data should be stored in medical records, along with how that data would be used, stored, and shared in the future-- considering the risks of unwanted identification-- are significant concerns, particularly in pediatric genomic sequencing, which poses a lifetime risk if performed in infancy or childhood [164]. Limitations of data privacy must be shared with the participants of genomic sequencing [178].

### 5.3. Increase the Accessibility to Genetic Tests

A survey of 162 neonatologists from 40 states and 112 NICUs in the USA showed that genetic consultations were only available in 78% of NICUs. WES or WGS were unavailable regularly in 69% of NICUs [179].

### 5.4. Develop Evidence-Based Clinical Practice Guidelines for Using WES or WGS in Critically Ill Infants with Suspected Genetic Diseases

The Medical Genome Initiative, a consortium of North American institutions formed to advance WGS into the clinic, has published best practices for analytical validation, measuring clinical utility, and interpretation and reporting for WGS [180,181,182,183].

However, evidence-based clinical practice guidelines for WES and WGS have only been developed by the ACMG for pediatric patients with congenital abnormalities or intellectual disability [184]. The ACMG guidelines were established using the Grading of Recommendations Assessment, Development, and Evaluation (GRADE) Evidence to Decision framework [185] to summarize the evidence and make healthcare recommendations [184]. These guidelines strongly recommend the use of WES or WGS as first-line or second-line tests in pediatric patients with congenital anomalies, developmental delay, or intellectual disability [184]. Nevertheless, evidence-based guidelines have yet to be developed for WGS or rWGS in critically ill infants with suspected genetic diseases or for pediatric patients with disorders other than congenital anomalies, developmental delay, and intellectual disability.

## 6. Limitations of rWGS in Critically Ill Children with Suspected Genetic Diseases

The limitations of rWGS include false-positive and false-negative results, challenges in variant interpretation and classification, cost and reimbursement barriers for widespread adoption, and limited availability of trained genetic counselors.

### 6.1. False-Positive and False-Negative Results

Genomic testing can lead to false-positive results due to several reasons [186,187]. Even with 99.9% accuracy in genomic sequencing, there are one million technical errors across a billion nucleotides [188]. Therefore, false-positive results may be seen in WGS despite high accuracy in sequencing [187]. Also, many literature-annotated variants are incorrect, incomplete, or common polymorphisms [189], indicating that false-positive results may also occur due to the erroneous assignment of a variant as pathogenic [187]. In addition, the immense number of variants generated by WGS can lead to clinical false positives regarding the clinical implications of the variant [190]. Diagnostic WGS differs from newborn screening (NBS) in that specific tests could be used to follow up after screening results [190]. Long-read sequencing and other emerging technologies hold promise for improving diagnostic WGS for clinical purposes [191,192].

Similarly, the complexity of the human genome can lead to false-negative results by WGS due to technical and analytic reasons [193,194]. Over two-thirds of the genome consists of repetitive sequences, which are not easily sequenced by short-read technology and lead to alignment challenges [193]. The accuracy of variants identified by WGS depends on the genomic region, variant type, read depth, and the analytical pipeline [193], with most false-negative WGS results occurring due to bioinformatic filtering. Systematic errors occur at some genomic locations using short-read technologies. Many such systemic sequencing errors have been called variants and included in variant databases [193].

### 6.2. Challenges in Variant Interpretation and Classification

Clinical laboratories adhere to guidelines established by the ACMG for assigning the clinical significance of germline genetic variants into five categories: benign, likely benign, VUS, likely pathogenic, and pathogenic [80]. However, discrepancies in variant classification are well-documented between laboratories [109,110,130,131,195,196,197]. The ACMG guidelines assign strength levels and include rules for combining evidence, including population data, allelic evidence, cosegregation data, computation and predictive data, and functional and other data, to classify any given variant [198]. The guideline structure is adaptable and facilitates data-sharing, which is essential for enhancing our understanding of the clinical significance of variants [199,200,201,202,203]. Discrepancies between laboratories have been resolved in an average of 17 days, even without inter-laboratory discussions, by comparing the laboratory findings with the structured evidence aspect of Australia’s “Shariant” platform [203]. However, this flexibility can also lead to inconsistencies in variant interpretation [198], which, unless resolved through discussions between laboratories [130,197,203], may result in unintended confusion in the diagnosis and clinical management of affected patients and their families. 

In addition to variant-level interpretations, case-level interpretations of variants must be integrated into the final clinical interpretation. This can be challenging due to the limited knowledge of penetrance and expressivity in many situations [198]. The ACMG guidelines are meant to be applied to Mendelian disorders with a relatively high penetrance. Therefore, caution is advised when applying these guidelines to secondary findings in asymptomatic or healthy individuals [198].

Furthermore, studies have shown that when variants are reclassified after a period following the initial diagnosis, the classification of VUS frequently shifts to the benign or likely benign category [204,205]. Previously diagnosed likely pathogenic variants have also been observed to change from likely pathogenic to VUS or from VUS to likely benign after a point-based system derived from the ACMG guidelines [206] had been applied to more than 500 variants with PM2 as the strength measure in the Shariant platform [203].

Moreover, WGS data include predominantly non-coding regions, which are not yet characterized regarding their functions in health and disease, except for known disease-causing variants in deep intronic and regulatory regions [35].

### 6.3. Costs and Reimbursement Barriers for Widespread Adoption

As discussed above, WGS can potentially reduce costs compared with standard genetic testing. A 2024 study in Canada and the U.K. investigated whether WGS for children with rare diseases can reduce healthcare costs [207]. Among 7775 patients in the 100,000 Genomes Project, 77 patients in a research setting, and 118 publicly reimbursed WGS recipients from Canada, no costs changed due to WGS [207]. In the U.K., 18.1% (*n* = 143) of children were diagnosed with epilepsy and 18.9% (*n* = 1323) with an intellectual disability by WGS. The mean annual per-patient spending in the U.K. over the study period, April 2021 to September 2023, was USD 5283 for epilepsy and USD 3373 for intellectual disability. In Canada, 54.5% (*n* = 42) and 39.8% (*n* = 47) of children in the research and publicly reimbursed settings, respectively, received a diagnosis by WGS, with costs of USD 724 in the research setting and USD 1573 in the reimbursed setting [207].

In the USA, WGS was estimated to cost USD 2094 per patient up to USD 9706 per trio, while WES costs were between USD 716 and USD 4817 per patient. These costs were based on seven studies published from 2016 to 2022 in Australia, Canada, the UK, the Netherlands (*n* = 2), France, and the USA. These studies provided costs for disaggregated resource use and labor, equipment, and consumables, with consumables comprising the main cost component [208].

In Australia, the costs of panel testing, proband-only and trio WES, and proband-only and trio WGS were reported in 2024 as follows [209]:

(1) Panel testing costs were AUD 2373 (AUD 733– AUD 6166).
(2) For proband-only analysis, WES costs were AUD 2823 (AUD 802–AUD 7206).
(3) For trio analysis, WES costs were AUD 5670 (AUD 2006–AUD 11,539).
(4) For proband-only analysis, WGS costs were AUD 4840 (AUD 2153–AUD 9890).
(5) For trio analysis, WGS costs were AUD 11,589 (AUD 5842–AUD 16,562).

Sequencing was the most expensive component of the costs, accounting for 36.9–69.4% of the total costs, while labor accounted for 27.1–63.2% of the total costs [209].

WGS has the highest costs, which, along with a lack of reimbursement in most parts of the USA and other countries without universal health coverage and accessibility, are significant barriers to implementing WGS in diagnostic clinical settings more widely.

### 6.4. Limited Availability of Trained Genetic Counselors

Trained genetic counselors are integral to providing pediatric genomic sequencing. As of 2018, nearly 7,000 genetic counselors were present in at least 28 countries. Only a few countries, including the USA, Israel, Norway, Canada, Australia/New Zealand, the UK, and Cuba, had 6–15 counselors per million persons [210]. There has been a growth of certified genetic counselors (CGCs) in the USA since 2013, when a task force was formed to increase the workforce due to a shortage [211]. In May 2017, there were 4242 CGCs in the USA [212]. Their practices in the USA are focused on cancer (52%), prenatal (41%), and pediatrics (29%) [213]. There is unequal distribution, with 683 practicing CGCs across the Southern U.S. in 2019 [213]. Most (98.7%) of CGCs (*n* = 4496) practice in metropolitan areas, with limited availability of trained genetic counselors in rural settings in the USA [214].

## 7. Newborn Screening by Genomic Sequencing

Traditional newborn screening (NBS) began in the 1960s with the work of Dr. Robert Guthrie in Buffalo, New York [215]. Today, NBS is usually offered as a public health service in high-income countries using dried blood spots from newborns [216]. The increased use of NGS has led to the hope of earlier detection of rare diseases using genomic sequencing, as reviewed [217]. In October 2023, 12 newborn sequencing (NBS) research programs from the USA, the UK, Europe, Australia, and the Middle East were discussed at an international conference in London, UK [218].

The most advanced of these NBS programs was the Genomic Uniform-screening Against Rare Diseases in All Newborns (GUARDIAN) study, which is being conducted in a diverse population in New York City [219]. This is a multisite, single-group, prospective, observational study of supplemental NBS by WGS with the goal of enrolling 100,000 newborns by approaching women in their third trimester of pregnancy who planned delivery at a participating hospital [218,219,220]. By March 2023, 74% (*n* = 1000) of the 1348 families approached had enrolled, showing a high rate compared to other NBS research studies. Most families consented in person at the bedside [219]. Between September 2022 and July 2023, 72.0% (*n* = 4000) of the 5555 families approached had consented to participate [220]. A targeted interpretation was conducted of WGS findings for 156 early-onset genetic conditions with established interventions selected by the investigators and 99 neurodevelopmental disorders associated with seizures if optionally chosen by the participants [220]. In most (90.6%) families, genomic NBS was performed for both groups of conditions; 9.4% of families consented only to be screened for disorders with established interventions. In the interim results published in January 2025, genomic NBS was positive in 3.7% of 3982 (99.6%) participants in whom sequencing was successful. The WGS screening results included 110 true positives for conditions not identified by traditional NBS [220]. The investigators returned VUS results in autosomal-recessive diseases only if they were accompanied by pathogenic and likely pathogenic variants and were predicted to be deleterious [220,221].

It is important to note that traditional NBS tests for the phenotypic manifestations of disease. In contrast, genomic NBS examines the genetic abnormalities that are predicted to cause the disease. A primary challenge of genomic NBS would be determining which variants identified by WGS are true positives and eliminating false negatives and false positives [222]. Other significant challenges include (1) genomic NBS across diverse racial and ethnic populations requires expanding the genomic variant databases, which currently include primarily individuals of European ancestry [223], (2) ethical dilemmas and challenges, which would need to be thought about while developing genomic NBS [223,224], and (3) privacy concerns and misuse of genetic data [223].

## 8. Conclusions

Within two decades of completing the Human Genome Sequencing, numerous researchers worldwide have worked to develop WES and WGS, including rWES and rWGS, to enhance genetic diagnoses and improve clinical outcomes for infants and children born with genetic diseases. Today, WGS can identify variants across the entire genome and can be performed rapidly, within hours, if deemed clinically necessary and urgent. As the costs of genomic sequencing continue to decrease and our understanding of germline variants in health and disease expands, WGS has the potential to become a first-tier clinical test for critically ill infants and children with suspected but undiagnosed genetic diseases. Rapid WGS should be prioritized in critically ill infants and older children with undiagnosed, suspected genetic diseases with phenotypes that are known to have a high probability of being diagnosed by rWGS and have the potential for specific treatment, including but not limited to neurodevelopmental disorders, cardiac abnormalities, and metabolic disorders. 

However, several critical issues must be addressed to enable the widespread use of WGS in clinical settings beyond established specialized centers. These include enhancing accessibility to genetic testing, educating non-genetic providers and parents, reducing costs, establishing cost-effectiveness in specific subsets of pediatric disorders, addressing various ethical challenges, and developing evidence-based clinical practice guidelines for the use of WGS in critically ill pediatric patients. Future studies should be prioritized to identify those subsets of pediatric patients for whom genomic sequencing, WES, or WGS would be beneficial and cost-effective for patients and families, promoting effective utilization of healthcare resources. This goal may involve using artificial intelligence methods to help target these rarely accessible and costly genetic tests to specific subsets of pediatric patients more widely.

## Figures and Tables

**Table 1:** Earliest studies for clinical exome and genome sequencing.

Publication Authors, Month, Year, Journal	Institutions, Studied Individuals or Cohort Characteristics	Clinical Sequencing Performed	Diagnoses by NGS or WES or WGS in N Probands	Significance
Ng et al. [39] September 2009, Nature	University of Washington, Seattle, USA, WES in 12 individuals: 4 unrelated with Freeman-Sheldon syndrome, 8 HapMap characterized	Whole exome	*MYH3* variants identified by WES	Proof of concept for the capability of WES to identify mutations in genetic diseases
Choi et al. [40] October 2009, PNAS	Multi-institutional, USA, and Turkey, exome sequencing in 6 patients, including 4 aged < 1-year, with a suspected diagnosis of Bartter syndrome	Whole exome	*SLC26A3* mutation in all 6 patients for a diagnosis of congenital chloride diarrhea	WES first applied to diagnose a genetic disease
Ng et al. [41], January 2010, Nature Genetics	University of Washington, Seattle, USA, WES in 4 individuals from 3 families with Miller syndrome, an autosomal recessive Mendelian disorder	Whole exome	*DHODH* variants identified as the cause; in 3 additional affected families, Sanger sequencing identified *DHODH* variants	WES in unrelated individuals identified the cause of rare genetic diseases
Lupski et al. [42], April 2010, NEJM	Baylor College of Medicine, USA, family with Charcot-Marie-Tooth Type 1 disease, with proband, 3 affected and 4 unaffected siblings, and unaffected parents; WGS performed in proband; sequenced exons 5 and 11 of *SH3TC2* for segregation analysis	Whole genome	*SH3TC2* (the SH3 domain and tetratricopeptide repeats 2 gene) dual mutations in proband and all affected family members	Characterized the molecular basis of a clinically diagnosed autosomal recessive genetic disease in a family
Hoischen et al. [43] June 2010, Nature Genetics	Radboud University Nijmegen Medical Centre, The Netherlands, WES in 4 affected individuals with Schinzel-Giedion syndrome	Whole exome	*SETBP1* mutations identified in all 4 individuals by WES, and in additional 8 affected individuals by Sanger sequencing	WES identified the molecular cause of an autosomal-dominant inherited disorder
Sobreira et al. [44] June 2010, PLOS Genetics	Johns Hopkins University School of Medicine, Baltimore, USA, WGS in a single patient with autosomal-dominant metachondromatosis (OMIM 156250) and partial linkage data from the proband’s family to identify the disease variant; also studied a 2nd family with the same disease and 469 control, unrelated individuals	Whole genome	Identified a frame-shift deletion in exon four of *PTPN11* in the proband, which co-segregated in all affected family members; in a 2nd family, identified a nonsense mutation in exon four of *PTPN11*; in 469 controls, showed the absence of any variants that predicted loss of *PTPN11* function	Characterized the molecular basis of the clinically diagnosed autosomal-dominant genetic disease in two families
Vissers et al. [45], November 2010, Nature Genetics	Radboud University Nijmegen Medical Centre, The Netherlands, trio WES on 10 individuals with moderate to severe intellectual disability with a negative family history, and unexplained cause	Whole exome	Identified de novo mutations in 9 genes, 6 of which in 6 individuals were considered to be likely pathogenic	Family-based WES identified the de novo nature of mutations in individuals with intellectual disability
Worthey et al. [46] March 2011, Genetics in Medicine	Children’s Hospital and Medical College of Wisconsin, Milwaukee, USA, early example of WES performed in a 15 mo. old male child with severe Crohn’s-like colitis non-responsive to treatment	Whole exome	Diagnosis of X-linked inhibitor of apoptosis (*XIAP*) deficiency; a hematopoietic stem cell transplant led to complete resolution of colitis	First WES diagnosis in a child with clinically unsuspected cause, led to specific curative treatment
Bainbridge et al. [47] June 2011, STM	Baylor College of Medicine, USA, WGS in a pair of 14 y old male and female twins, clinically diagnosed at age 5 y and treated for dopa-responsive dystonia (OMIM #128230) by L-dopa supplementation	Whole genome	Mutations in *SPR* encoding sepiapterin reductase reduced tetrahydrobiopterin, a co-factor for the synthesis of dopamine and serotonin; L-hydroxytryptophan (a serotonin precursor) supplement improved symptoms in both twins	Characterized the molecular basis of a clinically diagnosed genetic disease in fraternal twins
De Ligt et al. [48] October 2012, NEJM	Radboud University Nijmegen Medical Centre, The Netherlands, 100 individuals with intellectual disability underwent WES; confirmed the candidate genes in additional 765 individuals with intellectual disability	Whole exome	Diagnostic yield 16%; mutations: 10 de novo, 3 X-linked previously predicted for loss of function, and 3 in new candidate genes	WES effectively identified de novo mutations as the cause of intellectual disability
Saunders et al. [49] October 2012, STM	Children’s Mercy Hospital, Kansas City, USA, proof of concept rapid WGS in 7 acutely ill infants: retrospective WGS in 2 infants with known diagnoses (Tay-Sachs disease, Menke’s disease), prospective WGS in 5	Rapid WGS in 50 h	Diagnosis in 2/2 retrospective and 4/5 prospective WGS; in the 5th prospectively studied infant, WGS excluded known genetic diseases from the differential diagnosis	First demonstration of the diagnostic capability and clinical effectiveness of rapid WGS in acutely ill infants

NEJM, New England Journal of Medicine; PNAS, Proceedings of the National Academy of Sciences USA; STM, Science Translational Medicine; WES, whole-exome sequencing; WGS, whole-genome sequencing.

**Table 2:** Key cohort studies for clinical exome and whole-genome sequencing in children, 2013–2017.

Publications	Institutions, Country	Sequencing	Cohort Features	Infants in NICUs N (%)	Other Study Features	% (N) Probands Diagnosed by the Genomic Sequencing Test Performed ^a^	Other Features and Cost Savings, if Reported
Yang et al. [56], October 2013	BCM, USA	WES	250 unselected consecutive patients, 80% children with neurologic phenotypes; 50% (*n* = 124) ages < 5 y	No	October 2011–June 2012; prior genetic testing in all patients, including CMA, metabolic screening, and DNA sequencing tests	25% (62/250): 33 autosomal-dominant, 16 autosomal recessive, and 9 X-linked diseases, including 4 with two non-overlapping diagnoses; 33% in neurologic disorders	30 (12%)/250 had medically actionable incidental findings in 16 genes
Srivastava et al. [57], October 2014	JHU, USA	WES ^b^	78 patients (ages 1.6–26.3 y) from pediatric neurogenetics clinic with heterogeneous neurodevelopmental disorders	No	November 2011–February 2014; retrospective chart review for the diagnostic yield and utility of WES after non-diagnostic routine workup	WES diagnosis in 41% (32/78); affected clinical management in all 32 patients in different ways	
Yang et al. [58], November 2014	BCM, USA	WES	2000 patients; ages for 900 (45%) <5 y, 845 (42.2%) 5–18 y, 244 (12.2%) >18 y, 11 (0.6%) fetal; 87.8% neurologic disorders or developmental delay, 12.2% non-neurologic disorders	No	Observational study of consecutive, unrelated patients (88% pediatric) who underwent clinical WES June 2012 to August 2014	25.2% (504/2000); 36.1% in pediatric neurologic disorders; 280 (53.1%) autosomal-dominant, 181 (34.3%) autosomal-recessive, 65 (12.3%) X-linked, 1 (0.2%) mitochondrial; 5 (1%)/504 showed mosaicism for a mutant gene	92 (4.6%)/2000 had 95 medically actionable incidental findings
Lee et al. [59], November 2014	UCLA, USA	CES	814 consecutive undiagnosed patients, including 520 (64%) children with ages < 18 y; age < 5 y: 49% (254/520) of all children or 31% (254/814) of total *n*; 5–18 y: 32.6% (266/814)	No	17 January 2012–31 August 2014; trio WES in 50.3% (410/814), 74.8% (190/254) in ages < 5 y, 61.9% (163/266) in ages 5–18 y; WES proband only 41.5% (338/814), 73% (215/294) in ages > 18 y	26% (213/814); trio 31% (127/410) vs. 22% (74/338) proband-only WES; in ages < 5 y with developmental delay, trio WES diagnosed 41% (45/109) vs. 9% (2/23) by proband-only WES	5% cases with medically actionable incidental findings
Iglesias et al. [60], December 2014	CUMC, USA	WES	115 patients; 21 adults, 91 (78.9%) children with ages: 1–18 y, *n* = 83 (72.1%); 1–12 mo., *n* = 7 (6%), 0–30 days, *n* = 1 (0.9%), and 3 fetuses	No	WES October 2011 to July 2013; birth defects in 24.3%, developmental delay in 25.2%, seizures in 15%	32.2% (37/115); 53.5% in birth defects, 34% in developmental delay; post-WES, *n* = 8 screened for additional clinical features, *n* = 14 altered management, *n* = 2 given novel therapy	Canceled further testing in 100% after WES
Soden et al. [61], December 2014	CMH, USA	rWGS, retrospective	In 100 families, 119 children with neurodevelopmental disorders examined by parent–child trio WES (85 families) or rWGS (15 families) based on the acuity of the illness	Yes *n* = 15 (12.6%) from NICU and PICU; specific *n* for NICU unavailable	rWGS in families with neonates, infants, or children in ICU (in 50 h; mean coverage at least 30); trio WES in families with ambulatory children (in 16 days; mean coverage > 80) followed by WGS if WES non-diagnostic	Definite molecular diagnosis in 73% (11/15) families by rWGS and 43% (34/85) families with children in ambulatory clinics; in total, definite molecular diagnosis in 53 (44.5%)/119 affected children from 45/100 families; change in care in 49% of newly diagnosed families	USD 19,100 per family cost of prior negative tests in nonacute patients; sequencing was cost-effective at up to USD 7640 per family
Wright et al. [62], April 2015	DDD, UK	WES	1133 previously investigated yet undiagnosed children with developmental disorders; proband median age 5.5 y (range 1–16 y)	No	Trio WES in all; 987 (87%) children with intellectual disability, 270 (24%) had a history of seizures, and 121 (11%) had a congenital heart defect	27% (311/1133); trio WES reduced the number of variants that would have been flagged by proband only WES by 10-fold if neither parent was affected, and 3-fold or 1.5-fold if either parent was affected	
Willig et al. [63], May 2015	CMH, USA	rWGS, retrospective	35 families with infants <4 mo. old with an acute illness of suspected genetic cause underwent rWGS, which was retrospective compared with standard genetic tests performed earlier in 32 infants	Yes *n* = 35 (100%)	rWGS for trios, 11 November 2011–1 October 2014; median time to report 23 (range 5–912) d; 65% (13/20) rWGS diagnoses reported before discharge or death;	57% (20/35) by rWGS; 18/20 diagnoses not made by standard genetic tests, which diagnosed only 9% (3/32); acute clinical usefulness of rWGS in 13 (65%)/20, 4 (20%) diagnoses had strongly favorable CIM	Incomplete rWGS at death (*n* = 4); after rWGS results, 6 (30%) given palliative care
Taylor et al. [33], July 2015	WTC, UK	WGS	217 individuals from 156 individual cases or families with strong suspected genetic causes who underwent WGS; patient ages unavailable	No	From the WGS500 human genomes project initiated in 2010; earlier screening had not revealed a cause in these cases	21% diagnosed with causal variants, 34% (23/68) Mendelian disorders, 57% (8/14) in trios; WES would have missed 15% of the exonic causal variants	4 clinically actionable secondary variants in the 2013 ACMG gene list
Farwell et al. [64], July 2015	AG, USA	WES	500 clinical samples for WES; pediatric neurology disorders most frequent (65%) in cohort	No	September 2011 onwards, family-based exome sequencing; 338 trios, 21 duos, and 141 singleton WES	30% (152/500), 11 dual diagnoses; 37% in trios; 21% in singletons; highest rates: ataxia (44%), multiple congenital anomalies (36%), epilepsy (35%)	Trio WES found useful
Valencia et al. [65], August 2015	CCH, USA	WES	40 consecutive pediatric patients, all ages < 17 y at WES testing	No	Mean *n* genetic tests before WES = 4; previous CMA and single gene sequencing in 63%	WES diagnosis in 30% (12/40); medically actionable secondary findings in 3 genes in 3 of 36 patients	WES cost-effective and altered management
Miller et al. [66], September 2015	CMH, USA	rWGS in 26 h	Affected infants with a genetic condition nominated by a neonatologist; rWGS performed on research basis if suspected to have a diagnosis by NGS	No	rWGS version 2, in 26 h, with >99.5% analytical sensitivity and specificity (the 50-h version had >96.5% analytical sensitivity and specificity)	Applicable for selected medical conditions in NICU where rapid diagnosis could prevent significant morbidity and mortality	
Stavropoulos, et al. [67], January 2016	TSCH, Canada	WGS	100 clinic patients ages ≤ 18 y; abnormalities of the nervous system (77%), skeletal system (68%), growth (44%), eye (34%), cardiovascular (32%) and musculature (27%)	No	September 2013 to May 2014; WGS in all probands compared with standard clinical tests (CMA alone and CMA with targeted gene tests)	34% (34/100) by WGS, including copy number and sequence level variants; 8% by CMA, 13% by CMA and targeted tests (*p* = 0.0009); diagnosis rate for developmental delay higher by WGS, 38.6% (22/57)	Secondary variants by 2013 ACMG in 7 individuals
Sawyer et al. [68], March 2016	FORGE, Canada’s nationwide project	WES	>500 children from 362 families with childhood-onset rare disorders; most patients with a long diagnostic odyssey	No	Project outcomes: novel gene discovery and mutations in known disease-causing genes	Molecular diagnosis in known disease-causing genes in 29% (105/362) families or in a novel gene in 23% (83 families);	Clinical management changed in 6 (5.7%)/105 families
Retterer et al. [69], July 2016	GeneDx, USA	WES	3040 consecutive clinical cases at one clinical laboratory	No	January 2012 to October 2014; after May 2013, secondary findings analyzed in 2091 cases	28.8%, possible/probable 51.8%, a candidate gene diagnosed as a sole finding in 7.6%, negative 11.8%	Secondary findings in 6.2% (129/2091)
Daoud et al. [70], August 2016	CHofEO, Canada	NGS panel	20 newborns and infants in the NICU	Yes *n* = 20 (100%)	Studied by a panel of 4813 disease-relevant genes by NGS	40% (8/20) received a diagnosis based on the NGS panel analysis	
Stark et al. [71], November 2016	RCH, Australia	Prospective WES	80 infants (*n* = 62; <12 mo. age) and *n* = 18; 12–36 mo. age with suspected monogenic disease; no control group; SNP array pre-requisite for enrollment	Yes *n* = 33 (41%)	February 2014–May 2015, singleton WES evaluated as first-tier test with concurrent standard tests in the same cohort	57.5% (46/80) received WES diagnosis, compared with 13.75% (11/80) with standard tests alone; median time to report 134 (range 83–278) d	This study considered infants as having age > 12 mo. and <12 mo.
Trujillano et al. [72], February 2017	U of R, Germany	WES	1000 unrelated global patients with suspected Mendelian disorders; ages 1–5 y 39.4%, 5–15 y 28.5%, <1 y 14.1 %, 15–30 y 8.1%, >30 y 3.8%, prenatal 2.3%, consanguineous 45.3%	No	January 2014–January 2016; 54 countries: 78.5% middle East, 10.6% Europe, 5.8% S. Asia, 4.2% North and S. America; S. Africa 0.1%, Oceania 0.8%	30.7% (307/1000) diagnosed; 34.8% in consanguineous, 27.1% in nonconsanguineous families; several treatable diseases, metabolic diseases, and common diseases; dual diagnoses in *n* = 3	
Bowling et al. [73], May 2017	NACS, USA	WES, then WGS	371 individuals, ages > 2 y, with developmental delay/intellectual disability underwent genomic sequencing; replaced WES with WGS during the study	No	Trio 83.3%, duo 11.3%, singleton 5.4%, as part of the CSER consortium; WES in 365 individuals (127 affected) and WGS in 612 individuals (244 affected)	27% (100/371) pathogenic/likely pathogenic, including copy number variants, plus VUS in 11.3% (42/371); ACMG 2013 secondary findings in 12 (2%) parents; reanalysis of WES/WGS data improved diagnosis rate	WGS is an effective first-tier test, especially with parent-proband trios
Bick et al. [74], June 2017	CHofWI, USA	WGS	22 patients in genetics clinics underwent WGS	No	Pilot study during 2010–2013 after successful WES in a child with intractable IBD [46]	Initial diagnosis rate 14% (3/22) over 2 years; with re-analysis 33% (8/22)	
Stark et al. [75], August 2017	RCH, Australia	WES	Clinical cohort (*n* = 40; ages 0–36 mo.), the first 40 patients in [61]; prospective costs per patient, per diagnosis studied	Yes; *n* unavailable	Singleton WES and standard genetic tests performed in parallel in all patients for comparative purposes	57.5% by WES; WES more than tripled the diagnostic rate for one-third the cost (AUD 5047 or USD 3937 average) per diagnosis	Avg. AUD 27,050 or USD 21,099 for standard care
Vissers et al. [76], September 2017	RUMC, The Netherlands	WES	150 non-acute patients (age median 5 y, 7 mo., range 5 mo–18 y) with neurologic disorders, suspected genetic	No	November 2011–January 2015, WES and standard diagnostic tests	WES diagnosis in 29.3% (44/150), compared with 7.3% (11%) patients by standard diagnosis	Average cost EUR 3420 (EUR 744/patient less than standard)
Tan et al. [77], September 2017	MGHA, Australia	WES prospective	44 ambulatory children, ages 2–18 y, with a suspected monogenic disorder (mean age at initial presentation, 28 mo.; range, 0–121 mo.); singleton WES with a targeted phenotype analysis	No	May 1 to November 30, 2015; mean diagnostic odyssey 6 y; mean of 19 tests; 4 clinical genetics and 4 non-genetics specialist consultations for each child; 26 (59%) had a diagnostic procedure under general anesthesia	Diagnosis in 52% (23/44), unexpected in 35% (8/23); clinical management altered in 26% (6/23);	Cost savings AUD 9020 (USD 6838) per additional WGS tertiary visit diagnosis, and AUD 5461 (USD 4140) if WES at first genetics appointment vs. standard pathway
Van Diemen et al. [78], October 2017	UMC, The Netherlands	rWGS	23 critically ill infants (age median 28 d, range 1 day to 11 mo.) underwent singleton rWGS with targeted analysis for 3426 genes	Yes *n* = 23 (100%)	Prospective study May 2014–May 2016; rWGS report goal 14 d; parental DNA Sanger sequencing in 22/23 infants	Molecular diagnosis in 30% (7/23); report TAT median 12 (range 2–23) d; parental WGS not performed due to cost	
Meng et al. [79], December 2017	TCH, USA	Critical trio rWES	278 unrelated infants, ages < 100 d (median age 28 d), studied retrospectively by clinical WES: proband WES, trio WES, or critical trio WES (rWES)	Yes *n* = 190 (68.3%) in NICU; 43 (15.5%) in cardiovascular ICU; 18 (6.5%) in PICU	December 2011–January 2017; 178/278 singleton, 37/278 trio, 63/278 critical trio rWES; CMA in 85% (237/278); median TAT 13 d critical rWES, 95 d proband-only, 51 d trio	WES diagnosis in 36.7% (102/278) infants; 50.8% (32/63) diagnosis by critical trio rWES (*p* = 0.011226) higher than proband WES (32.6%, 58/178) or trio WES (32.4%, 12/37)	CIM in 72% of critical trio WES vs. 43% of regular WES

The seven rows with a light blue or light gray (cohort *n* < 25) background depict studies involving infants in NICUs. The single light green-colored row represents a study that included children in PICUs; all remaining colored or white rows represent studies involving older children in non-acute clinical settings. The beige-colored rows and rectangles represent trio WES or trio WGS studies. Abbreviations: ACMG, American College of Medical Genetics and Genomics; AG, Ambry Genetics, clinical reference laboratory; BCM, Baylor College of Medicine; CCH, Cincinnati Children’s Hospital; CES, clinical exome sequencing; CHofEO, Children’s Hospital of Eastern Ontario and Ottawa Hospital; CHofWI, Children’s Hospital of Wisconsin in Milwaukee; CIM, changes in management; CMA, chromosomal microarray; CMH, Children’s Mercy Hospital, Kansas City; CSER, Clinical Sequencing Exploratory Research; CUMC, Columbia University Medical Center; d, days; DDD, Deciphering Developmental Disorders national study; FORGE, Finding Of Rare Disease Genes; GeneDx, GeneDx clinical reference laboratory; IBD, inflammatory bowel disease; ICU, intensive care unit; JHU, Johns Hopkins University; MGHA, Melbourne Genomics Health Alliance; NACS, North Alabama Children’s Specialists, Huntsville; NICU, neonatal intensive care unit; NGS, Next generation sequencing; PICU, pediatric intensive care unit, RCH, Royal Children’s Hospital, Melbourne; RUMC, Radboud University Medical Center; rWES, rapid WES; rWGS, rapid WGS; S., South; SNP, single-nucleotide polymorphism; TAT, turnaround time; TCH, Texas Children’s Hospital; TSCH, The Sick Children’s Hospital, Toronto; UCLA, University of California at Los Angeles; U of R, University of Rostock; UMC, University Medical Center, Groningen; VUS, variants of uncertain significance; WES, whole-exome sequencing; WGS, whole-genome sequencing; WTC, Wellcome Trust Center for Human Genetics, University of Oxford; ^a^ Considered as diagnosed if pathogenic or likely pathogenic variants per ACMG criteria [80]; ^b^ WES performed by outside laboratories.

**Table 3:** Key studies for exome and genome sequencing, 2018 to 2024.

Publications	Institutions, Country	Study Type	Cohort Features	Infants in NICU *n* (%)	Other Study Features	Diagnoses by NGS, WES, WGS, rWES, or rWGS in N Probands	Additional Characteristics, Including Cost Savings, if Reported
Petrikin et al. [88], February 2018	RCHSD, USA	NSIGHT1 RCT for rWGS	65 infants < 4 mo. age in NICU (*n* = 64) and PICU (*n* = 1) suspected to have genetic diseases randomized into 2 groups, control with standard tests (*n* = 33), the other with standard tests and rWGS (*n* = 32); all infants received newborn screening	Yes 65 (100%)	October 2014 to June 2016, follow-up until November 2016; 63 infants received average 3.1 (range 0–10) standard genetic tests, including panel NGS, WES, and WGS in 73% (24/33) controls and 53% (17/32) rWGS groups; primary endpoint: 28-day molecular diagnosis rate	31% (10/32) by rWGS, 28%> than by standard tests alone (3%, 1/33), *p* = 0.003; crossover requested in 21% (7/33) controls and granted in five; 2 of those 5 diagnosed by rWGS	Median time to result about 5 days for rWGS, for well-covered SNVs in high quality WGS regions; confirmatory Sanger sequencing for rWGS results in all patients added 7–10 days to report diagnoses in this trial
Farnaes, et al. [89], April 2018	RCHSD, USA	rWGS	42 acutely ill admitted infants, age <1 y, with no etiologic diagnosis in whom a genetic disorder was possible evaluated by rWGS; retrospectively studied	Yes 42 (100%)	26 July 2016, to 8 March 2017: 29 trios, 1 quad (parents and 2 affected siblings), 9 mother–infant duos, 3 singletons; concurrent 144 standard genetic tests performed in 79% (33/42) infants	43% by rWGS (18/42, including 3 partial diagnoses vs. 10% by standard tests; clinical utility 31% (13/42) by rWGS vs. 2% by standard tests	In *n* = 6, inpatient stay reduced by ~124 days with USD 803,200 professional and facility costs; average cost savings USD 19,000 per infant (*n* = 42) sequenced by rWGS
Lionel et al. [90], April 2018	TSCH, Canada	WGS and WES, research-based	103 patients, ages ≤ 18 y, diverse phenotypes from pediatric subspecialty non-genetics clinics with suspected genetic disease	No	April 2013 to June 2015; prospectively compared research-based proband WGS with standard clinical genetic tests, and WGS with WES in the first 70 patients	41% (42/103) by WGS, higher (*p* < 0.01) than 24% (25/103) by standard testing	18 WGS diagnoses in 17/42 included structural and non-exonic variants not detectable by WES
Clark et al. [91], July 2018	Meta-analysis	Meta-analysis	Systematic review of articles published January 2011 to August 2017 for the utility of WES, WGS, and/or CMA	No; these studies were for older children	Among 2093 studies identified by literature searches, 37 studies analyzed with a total of 20,098 children	Diagnostic utility of pooled WES: 0.36; WGS: 0.41, and CMA: 0.10; WGS utility > CMA, *p* < 0.0001	WES or WGS suggested as a first-tier genetic test with greater diagnostic utility than CMA
Mestek-Boukhibar et al. [92], November 2018	UCL, U.K.	rWGS in PICU	24 critically ill children in PICU, age median 2.5 mo. (range 7 d–13 y 2 mo.); trio rWGS nomination by subspecialty consultants	Yes 24 (100%)	Developed a multidisciplinary workflow, including phased analysis, in the first 10 patients, applied to the next 14 patients	42% (10/24), all in the first phase; immediate impact on management in 30% (3/10) diagnosed patients	Shortest time to provisional diagnosis 4 calendar d for the last 14 patients, median time 8 d
Stark et al. [93], December 2018	2 CHs, Melbourne, Australia	rWES	40 children (ages 0–18 y) with likely monogenic disease underwent rapid singleton WES	Yes 21 (53%)	Compared cost with previous cohort of standard WES [65], median TAT 16 (range 9–109) days	52.5% (*n* = 21/40) by rWES, outperformed biochemical tests in 2; CIM in 12 (57%)/21 diagnosed	Cost per diagnosis of rWES: AUD 13,388 (USD 10,453), standard WES AUD 10,843.60
French et al. [94], March 2019	NHS CUS, U.K.	WGS	Prospective WGS in 195 probands (106 NICU, 61 PICU, 28 pediatric neurology or clinical genetics) and their families; median age in NICU 12 days (range 1 day–6 mo.)	Yes 106 (54%)	December 2016 to September 2018: 90% trios, 9% duos, 1% singleton; non-NICU median age 24 mo. (range 8 d to 16.8 y), and one 23 y old patient; broad inclusion criteria for suspected single gene disorders	21% (40/195) diagnosed: 13% NICU, 25% PICU, 39% pediatric neurology or clinical genetics; ages of diagnosed probands 1 day–15 y; higher diagnosis rates in specific phenotypes in NICU and PICU	2–3-week TAT sufficient for most clinical decision making; 50% of eligible parents declined or did not respond to WGS offer
Clark et al. [95], April 2019	RCHSD, USA	rWGS	375 symptomatic children underwent rWGS in <21 h from test request to diagnosis	Yes *n* unavailable	By automated CNLP for phenotype extraction from electronic health records, combined with automated pipeline for WGS	27% (*n* = 101) diagnosed by rWGS with 105 genetic diseases; 274 children did not receive a diagnosis by WGS	Median 20:10 h for WGS genetic diagnosis by CNLP-extracted features and automated pipeline
Kingsmore et al. [96], October 2019	RCHSD, USA	NSIGHT2 RCT: ultra-rWGS, rWES, rWGS	578 (46%)/1248 seriously ill infants age < 4 mo. and <96 h from admission or development of abnormal symptoms eligible; 213 enrolled; 670 ineligible infants included 33 with previously confirmed genetic diagnoses	Yes 213 (100%)	29 June 2017, to 9 October 2018 (467 days), 24/213 severely ill infants received ultra-rWGS, remaining 189 randomized to receive rWES (*n* = 95) or rWGS (*n* = 94); 69% (147/213) families with trio samples	24% (52/213) with 55 genetic diseases; 46% (11/24) by ultra-rWGS vs. 20% (37/189) randomized, *p* = 0.004; explained symptoms completely in 87% (*n* = 45), partly in 4% (*n* = 4); 6 (11%) incidental or actionable findings	Minimum incidence of genetic diseases in infants ages < 4 mo. in NICU, PICU, and cardiac ICU at least 14% [{(52 × 578/213) + 33}/1248]; More than half of eligible families denied consent for research WGS
Lindstrand et al. [34], November 2019	KUH, Sweden	WGS	First 100 clinical genetics cases referred for CMA in 2017; first validated the SV calling pipeline by short-read WGS, then prospectively analyzed by WGS in parallel	No	WGS data were processed for large SVs (>10 kb), genome-wide and small SVs (>2 kb), and for SNVs and INDELs in 887 genes linked to intellectual disability	WGS diagnosis in 27%, compared with 12% by CMA; derivative chromosomes with additional complexities were detected and resolved by WGS	WGS detected all known SVs and identified additional SVs; WGS can be used as first line single test in intellectual disability instead of CMA and WES
Sanford et al. [97], November 2019	RCHSD, USA	rWGS in PICU (outside of NICU)	38 children in PICU > 4 mo. age (median 2.96 y, average 5.73 y, range 4 mo. to 17 y) underwent rWGS; 74% nominated by intensivists; retrospective study	Not in NICU	July 2016 to May 2018: 24 trios, 4 parent–child duos, 10 singletons; patient characteristics and outcomes studied (changes in PICU and non-ICU clinical management, palliative care decisions and family screening)	42% Hispanic/Latino patients; genetic diseases diagnosed in 45% (17/38); CIM in ICU 24% (4/17), non-ICU 65% (11/17); no change 12% (2/17) due to rWGS	TAT sample receipt to report averaged 13.6 d (range 1–56 d), variable in 2016 and early 2017 due improving workflow and pipeline; cost efficacy described in Jan 2022 [98]
Gubbels et al. [99], April 2020	BCH, USA	rWES trio in <7 days	50 infants, age <6 mo. with specific phenotypes: hypotonia, seizures, a complex metabolic phenotype, and/or MCA in *n* = 49; *n* = 1 with DSD	Yes *n* = 43 (86%) in NICU, and *n* = 7 in other ICUs	March 2017 to November 2018; most common included phenotype: MCA (*n* = 37); mitochondrial DNA also sequenced by NGS in *n* = 16, with diagnosis in *n* = 1	29 (58%)/50 diagnosed by rWES (80% if hypotonia, 90% if seizures): 27 known, 2 novel genes; CIM in 24 (83%)/29; 13 (26%)/50 died, including 10 (33%)/30 diagnosed	In 57 families identified for study, 5 declined due to timing conflicts with clinical care (*n* = 4) and privacy concerns (*n* = 1), and 2 withdrew after enrollment
Wang et al. [100], May 2020	CH of FU, China	rWES trio in 24 h	24 h trio rWES validated in 33 critically ill infants (ages 10/33 ≤ 28 d; 23/33 > 28 d; median 51 d, range 2–210 d); all 33 infants underwent WES and rWES	Yes 33 (100%)	May–June 2018; 27% (128/473) patients in PICU/NICU with suspected genetic diseases; 40 infants with complex undiagnosed diseases met criteria (7 parents refused enrollment)	69.6% (23/33) diagnosed by rWES (ages in 7/10 ≤ 28 days, 16/23 > 28 days) and in additional 2/33 by WES; specific changes in treatment in 43.5% (10/23)	TAT rWES median 24 h (22–27 h); regular WES 10 days (9–12 days); rWES cost USD 2200, equivalent to the fee for a 2–3-day ICU stay; regular WES cost USD 1500
Australian GHA [101], June 2020	Australian GHA	Ultra-rWES	108 critically ill infants and children, age median 28 d (range 0 d–17 y), from NICU (*n* = 62), PICU (*n* = 36), other hospital in-patients (*n* = 10)	Yes 62 (57%)	Prospective study, March 2018 to February 2019 for ultra-rWES (results in 5 d), data collected until May 2019; 7 (6%) underwent concurrent mitochondrial genome testing	51% (55/108) diagnosed: 56% (35/62) NICU, 47% (17/36) PICU, 30% (3/10) other admitted patients; see [101] for CIM	Sample to report median time 3 (2–7) d; 94% (*n* = 102) reports <5 d; compared with their previous rWES study that resulted in 21 d [93]
Freed et al. [102], June 2020	SCH, USA	rWES	46 patients (median age 25 d, age range 1 day to 15 y) with suspected genetic disorders in NICU (56%), PICU (22%), and cardiac (22%) ICU	Yes 56% in NICU	October 2016 to July 2019, trio rWES at a send out laboratory, GeneDx, as a first-tier test in 46% (21/46), and second-tier test in 54% (25/46) after CMA and other gene tests	43% (20/46); plus 8.6% (4/46) partial diagnosis; 5 deaths despite rWES, 2/5 diagnosed after death; CIM in 52% (24/46, 19 diagnostic, 5 non-diagnostic)	WES TAT 5–26 (median 9) d, included 3-d committee review, pre-test counseling, trio sample collection; counseling challenging in *n* = 4 for secondary findings [103]
Smigiel et al. [104], July 2020	WMU, Poland	rWES	18 critically ill infants in ICU (10 NICU, 8 PICU) and families who consulted genetics specialists	Yes 18 (100%)	During 2015–2018; infants underwent rWES as a first-tier test (*n* = 9) and after other genetic tests (*n* = 9)	72.2% (13/18) infants diagnosed, including 8/13 inborn errors of metabolism; 88.9% diagnoses in first-tier rWES	TAT 5–14 days; 77.7% (14 of 18) infants died; WES results for 7 infants received after death
Dimmock et al. [105], November 2020	RCHSD, USA	NSIGHT2	NSIGHT2 RCT [96], described above in this table; clinicians provided input in 94% (*n* = 201) infants	Same as NSIGHT2 RCT [96]	Evaluation of the clinical utility, changes in management, and perceived harm by physician surveys for the NSIGHT2 RCT	Diagnostic sequencing useful or very useful in 77% (154/201)	High clinical utility, low perceived harm, parental perceptions described in separate publication [106]
Stranneheim et al. [107], March 2021	GMCK-RD, Sweden	WGS	4437 individuals (3219 patients and 1218 relatives) underwent WGS; 84% singleton, 16% trio/family analyses	No	Mid-2015 to 2019; clinical genomics workflow included phenotype-specific gene panels and an OMIM morbid gene panel for patients with complex phenotypes	40% (1285/3219) diagnosed (19–54% in specific disease groups); singletons 34%, trios 36%; 35% disease-specific panels vs. 41% for OMIM morbid gene panel	3 groups of TAT analyses (from sample receipt to report) based on clinical urgency: Regular 1–3 mo., priority 2–4 weeks, acute TAT 4–14 days
Sweeney et al. [108], April 2021	RCHSD, USA	rWGS	Acutely ill infants < 1 y age (*n* = 31) referred with congenital heart disease symptom onset in the neonatal period; *n* = 24 underwent rWGS	20 (83%) from NICU; 3 (13%) from cardiac ICU	July 2016–June 2017: trio in 16, singleton in 5, duo in 2, quad in 1; both rWGS and CMA performed in 19 of 24 infants	46% (11/24) by rWGS; clinical genetic tests 10% (2/24); among 19 with both rWGS and CMA, 7 (37%) by rWGS, 1 (5%) by CMA	4 referred families declined rWGS after consent process; CIM in all patients led to decreased hospitalization cost
Maron et al., May 2021 [109], and July 2023 [110]	Multi-site, 6 hospitals in the USA	rWGS GEMINI trial	Prospective multi-year trial from June 2019 to Nov 2021, enrolled 400 hospitalized infants < 1 y age with a suspected undiagnosed genetic disorder and parents (388 mothers; 318 fathers); interim report [109] and final results [110]	Not in NICU	Targeted genomic sequencing of 1722 actionable genes for disorders that present in the first year of life at Quest laboratories compared with rWGS at RCH, with report in 14 days in both platforms; ultra-rWGS in urgent cases with report in 72 h; trio testing preferred	51% (204/400) molecular diagnoses; 49% by rWGS median 6.1 days (routine rWGS) vs. 27% in median 4.2 days by targeted gene sequencing; ultra-rWGS quicker in 3.3 d; 46% discordance in variant interpretation in the two laboratories	Changes in clinical care in 19% participants, 6% (24 variants in 374 probands) actionable secondary findings; 3 mothers and 1 father informed of carrying variants with increased cancer risk
Dimmock et al. [111], July 2021	Project Baby Bear, Califonia, USA	rWGS or ultra-rapid trio WGS	rWGS broadly applied in newborns in 5 hospitals in California, enrolled 184 acutely ill infants < 1 y age who were beneficiaries of the state Medicaid program; included 55% Hispanics, a historically underserved population	Not in NICU	Admitted November 2018 to May 2020 with no clear non-genetic etiology, hospitalized ≤ 1 week or an abnormal response to standard therapy for an underlying condition in the preceding week; clinical rWGS in all, or if deemed too unstable to wait, ultra-rapid trio WGS	40% (74/184) diagnosed; 32% (58/184) CIM; 31/58 had substantial changes in inpatient stay and medical procedures; VUS in 11% (21/184); no diagnosis 48% (89/184) infants; TAT median 3 d for provisional report	Only 6 families denied consent; USD 9492 rWGS cost/child; USD 2.2- USD 2.9 million decrease in hospital costs and professional fees for *n* = 31; in *n* = 184, avg. USD 12,041 to USD 15,786 savings per child sequenced by rWGS
Wu et al., [112], October 2021	13 hospitals in 10 provinces in China	rWGS	202 critically ill infants < 13 mo. age, 61% males, with suspected genetic disease: {51.5% (*n* = 104) <28 d, 31.2% (*n* = 63) 28 d to 3 mo., and 17.3% (*n* = 35) 3–13 mo. age	Yes 202 (100%)	China Neonatal Genomes Project, April to December 2019; neuromuscular (45%), respiratory (22%), immunologic/infectious (18%) phenotypes most common	36.6% (*n* = 74; *n* = 45 for ≤28 d, *n* = 19 for 28 d to 3 mo., *n* = 10 for ≥3 mo.) diagnosed by trio-rWGS in median 7 days vs. 20.3% by proband-only CES in median 20 days	Targeted treatments in 21.6% (16/74); 32.4% (24/74) referred to subspecialists; rWGS cost higher vs. singleton CES but reduced hospitalization duration/costs
Krantz et al. [113], December 2021	5 medical centers/CHs, USA	NICUSeq trial sponsored by Illumina; WGS	354 ICU infants 1–120 d old with suspected genetic disease; observed until 2 July 2019; randomized for WGS results reported at 15 d (*n* = 176, early group) or 60 d (*n* = 178, delayed group), observed for 90 d	Yes 354 (100%)	Enrolled 11 September 2017–30 April 2019; trio (74%), duo (23%), or quad (3%) WGS in Illumina’s clinical laboratory; 83% (296/354) NICU, 7% (23) PICU, 10% (35) infants from cardiac ICU	9% (32/354) died; early group diagnosis rate 31% (55/176), which was 2-fold compared to the delayed group at 60 days; delayed group diagnostic rate also 31% (56/178, two-fold increase after WGS) at 90 d	35% of approached families declined WGS; CIM 2-fold > in early group at 60 d, and in delayed group at 90 d; COM in 24% (45/182) congenital anomalies, and 35% (17/45) neurologic disorders
Bowling et al. [114], April 2022	Multi-site, 5 clinical centers in Southeastern USA	SouthSeq clinical research study, WGS	367 admitted infants (age median 14, range 0–379 d) in 365 families; NICU, PICU, surgical or cardiac ICU with suggested genetic abnormalities or unexplained major medical disorders, including seizures and metabolic abnormalities; gestational age of probands median 36 (range 22–42) weeks	Yes 367 (100%) in an ICU, *n* for NICU unavailable	Admitted February 2018 to July 2020, 74% families racial/ethnic minorities; 86% also received standard tests; singleton WGS performed in a clinical laboratory, with variants analyzed by a research protocol, parental samples (234/367 trios, 104/367 duos) used for Sanger sequencing confirmation of clinically relevant variants	44% probands with WGS variant for the test indication: 30% definitive/likely diagnosis and 14% uncertain; diagnostic rates did not differ in Black/African and White/European Americans, and among the study sites; 57% WGS results not identified by standard testing	6 infants had 7 pathogenic/likely pathogenic secondary variants; educated non-genetic providers in returning sequencing findings [115]; parents considered neonatal WGS to be useful [116] and showed interest in genetic ancestry results from clinical providers over commercial entities [117]
Denommé-Pichon et al. [118], May 2022	France, 8 national reference centers	rWGS FASTGENOMICS study	37 infants (23 males, 14 females) in NICU or PICU suspected of genetic disease, median age of probands 27 days	Yes 37 (100%) NICU and PICU	French prospective pilot study from December 2018 to February 2020; prioritized WGS for infants in ICU in the diagnostic process nationally	49% (18/37) showed causal variants and 22% (8/37) VUS; median TAT 42 days achieved after corrections in first phase	Only urgent cases underwent rWGS to control costs: 7900 € per trio rWGS vs. 2590 € routine workflow
Bupp et al. [119], January 2023	Project Baby Deer USA	rWGS	89 critically ill neonatal and pediatric inpatients, ages < 18 y, in the state of Michigan [120]	Yes NICU and PICU, specific *n* unavailable	rWGS performed at RCH, like Project Baby Bear; led to Medicaid insurance coverage for rWGS for inpatients with ages < 1 y in Michigan	39% (*n* = 35) diagnosed; CIM in 27% (*n* = 24)	95–214 hospital days avoided, net savings USD 4155 per patient, family experience improved
Lumaka et al. [121], February 2023	Belgium	rWGS	21 critically ill children in the NICU (*n* = 9), PICU (*n* = 6), or the neuropediatric unit (*n* = 6)	Yes 9 (42.8%)	rWGS as a first -tier test in all; average TAT of 39.80 h (range 37.05–43.7 h)	12 (57.5%) diagnosed {5/9 NICU, 4/6 PICU, 3/6 neurologic}, all defects were SNVs	CIM in all 12 diagnosed patients
Olde Keizer et al. [122], June 2023	The Netherlands	rWES; RADICON-NL consortium	60 neonates in 5 Dutch NICUs with suspected genetic diseases, prospective study;	Yes 60 (100%)	May 2017 to January 2019: rWES and routine genetic testing performed in parallel on all probands	20% by rWES in 15 d (shorter time), than 10% by routine tests in 59 d	rWES reduced genetic diagnostic costs by 1.5% (€85 per neonate)
Marouane et al. [123], January 2024	The Netherlands	rWES	298 children (ages < 4 weeks, *n* = 114; 1–23 mo., *n* = 115; 2–5 y, *n* = 36; 6–12 y, *n* = 20; 13–18 y, *n* = 13); 241 prenatal, 36 adults (total *n* = 575)	No	85% trios, 14% singleton, <1% duo; <1% quartet; median rWES TAT 11 d (range 8 to 15 d)	30.4% in 175/575 (31.0% in <18 y and 22.2% in ≥19 y); SNV/indels in 89.1% (*n* = 156), CNVs in 10.3% (*n* = 18)	rWES was helpful across all patient ages and in the broad spectrum of rare diseases in non-acute clinical settings
Marom et al. [124], February 2024	Israel, nationwide	rWGS	130 neonates (54% male, 46% female) in NICUs (*n* = 25) received trio rWGS at TASMC Genomics Center; prospective national pilot study	Yes 130 (100%)	October 2021 to December2022; mean (SD) TAT 7.4 (2.7) days to rapid reports, 67.6 (26.1) days to secondary analysis; 6.5 (2.3) for causative variants vs. 8.5 (3.3) for negative variants	50% (*n* = 65) diagnosed, most commonly exonic SNVs followed by chromosomal abnormalities; negative results in 38% (*n* = 50)	rWGS was feasible and diagnostically beneficial in critically ill neonates in a public healthcare setting
Thompson et al. [125], February 2024	Multi-site, 8 hospitals in USA	rWGS	188 admitted infants in ICU or general floor; 60% male, all <1 y age with suspected genetic disease; retrospective study	Yes *n* in NICU unavailable	November 2017 to April 2020; rWGS performed at RCH, HPO-driven interpretation of rWGS results; average TAT 6 d (vs. 11–15 d NSIGHT and NICUSeq)	35% positive diagnoses, 49% negative results, 12% VUS, 4% had incidental findings, shorter hospitalization in 20%	32% major CIM (medication, diet, surgery, palliative care), 40% minor CIM: additional genetic counseling
Sloper et al. [126], April 2024	Wales, U.K.	Trio rWGS	82 families with acutely unwell infants and children aged 2 d to 16.69 y; 74% ages < 1 y	Yes *n* in NICU unavailable	2020 to 2023, Wales Infants’ and children’s Genome Service (WINGS); mean TAT 9 days	34.1%; highest rates in skeletal dysplasia, neurological or metabolic phenotypes	Positive impact of rWGS on pediatric health in the National Health Service (NHS) setting
Migliavacca et al. [127] June 2024	Brazil	Trio WGS	21 critically ill infants with dysmorphic syndromes, metabolic errors, and skeletal dysplasias	Yes 21 (100%)	Infants hospitalized in NICUs in the Brazilian healthcare system; trio WGS TAT 53 days	57% (*n* = 12/21); 16 pathogenic/likely pathogenic variants and 10 VUS	Utility of WGS as a diagnostic tool outperforming standard genetic tests
Wojcik et al., [35], June 2024	BCH, USA	WGS	744 families in initial cohort, proband age median 12 y (range 5 y–36 y); additional 78 families cohort; previous WES non-diagnostic in 474 (63.7%) of 744 and 51 (65.3%)/78 families	No	April 2016 to March 2021 for the initial cohort, and 2018 to 2022 for additional cohort; WGS: proband-only, duo, trio, and larger family groups	Definite or probable molecular diagnosis by WGS in 218 (29.3%)/744 and 18(23%)/78 families; known genes in 72% (*n* = 157/218) and novel genes in 27.5% (*n* = 60/218) families	28.0% (*n* = 61/218) and 33% (*n*= 6/18) of diagnosed cases required WGS; the causal variants: deep intronic or complex structural variants or tandem repeat expansions would be unlikely to be identified by typical WES
Rodriguez et al. [128], August 2024	Four CHs, USA	rWGS in PICU or cardiac ICU	133 patients 0–18 y, median (IQR) age 6 mo. (IQR 1.2 mo–4.6 y), in PICU or cardiac ICU	No; NICU stay was an exclusion criterion	2016–2023; 97 retrospective and 36 prospective; most common clinical features: cardiac (31%), neurologic (24%), primary respiratory (15%), dysmorphism (11%)	Molecular diagnosis in 59% (*n* = 79); dysmorphic features and congenital heart disease higher odds of diagnosis	Diagnosis rate unaffected by the specialist ordering rWGS

Studies including infants in NICUs are depicted in rows with a light blue or light gray (cohort *n* < 25) background color. The three light green-colored rows represent studies of children in PICUs, not in NICUs. All remaining colored or white rows represent studies that included older children, including in non-acute clinical settings. The light beige colored rows and rectangles represent studies that used trio WES or trio WGS. Abbreviations: BCH, Boston Children’s Hospital; CES, clinical exome sequencing; CHs, Children’s hospitals; CH of FU, Children’s Hospital of Fudan University; CIM, changes in management; CMA, chromosomal microarray; CNLP, clinical natural language processing; DSD, disorder of sex development; GEMINI, Genomic Medicine in Ill Neonates and Infants trial; GHA, Genomics Health Alliance (12 hospitals and 2 laboratories); GMCK-RD, Genomic Medicine Center Karolinska-Rare Diseases; HPO, human phenotype ontology; ICU, intensive care unit; INDELs, insertions and deletions; IQR, inter-quartile range; KUH, Karolinska University Hospital; MCA, multiple congenital abnormalities; NGS, next generation sequencing; NHS-CUS, National Health Service Cambridge University Hospitals; NICU, neonatal intensive care unit; NSIGHT2, Newborn Sequencing in Genomic Medicine and Public Health-2; PICU, pediatric intensive care unit; RCH, Rady Children’s Hospital; RCT, randomized control trial; rWES, rapid WES; rWGS, rapid WGS; SCH, Seattle Children’s Hospital; SD, standard deviation; SNVs, single-nucleotide variants; SV, structural variant; TASMC, Tel-Aviv Sourasky Medical Center; TAT, turnaround time (time to diagnosis); TSCH, The Sick Children’s Hospital, Toronto; UCL, UCL Great Ormond Street Institute of Child Health, London; WES, whole-exome sequencing; WGS, whole-genome sequencing; WMU, Wroclaw Medical University.

**Table 4:** A comparison of rWGS with WES and targeted panel NGS in pediatric genomic sequencing.

Variables	rWGS	WES	Targeted Panel NGS
**The genomic regions interrogated**	Almost all coding and non-coding regions of the genome	Only the protein-coding regions (exons) of all genes, comprising 1–2% of the entire genome	Variable numbers of genes in various gene panels, which are usually targeted to a specific disease
**Cost**	Highest; constantly declining	Intermediate	Lowest
**Turnaround time**	Varies from <24 h in ultra-rapid WGS to a few weeks for the final report for rWGS, based on the institution’s protocol	Usually weeks, unless rWES, based on various factors, including where performed, accessibility, and send-out test or not	Few days to weeks, depending on various factors, including where the test is performed, accessibility, and send-out test or not
**Accessibility**	Limited to specialized institutions	Greater than rWGS but still limited to specialized centers and commercial laboratories	Most accessible
**Diagnostic yield**	Highest among all genomic sequencing procedures; can identify novel disease-causing variants	May be helpful in some instances but does not identify many types of variants; diagnostic yield usually lower than WGS	May be helpful if the patient is suspected to have the specific disease targeted by the disease-focused panel
	May be higher with trio sequencing than proband-only sequencing	May be higher with trio sequencing than proband-only sequencing	Not applicable
**Clinical Utility**	Can be very useful in diagnosing some phenotypes, including in those patients with a missed diagnosis by WES, leading to clinical management changes	Not as useful as WGS in suspected but undiagnosed genetic diseases	Useful if a known disease in a family is being tested for in a member of the same family; not useful in suspected but undiagnosed genetic diseases

Abbreviations: NGS, next-generation sequencing; rWGS, rapid whole-genome sequencing; WES, whole-exome sequencing.

## Abbreviations

The following abbreviations are used in this paper:

ACMG, American College of Medical Genetics and Genomics; AG, Ambry Genetics clinical reference laboratory; AUD, Australian dollar; BCH, Boston Children’s Hospital; BCM, Baylor College of Medicine; CCH, Cincinnati Children’s Hospital; CES, Clinical exome sequencing; CGCs, certified genetic counselors; CHofEO, Children’s Hospital of Eastern Ontario and Ottawa Hospital; CHofWI, Children’s Hospital of Wisconsin in Milwaukee; CHs, Children’s hospitals; CH of FU, Children’s Hospital of Fudan University; CIM, changes in management; CMA, chromosomal microarray; CMH, Children’s Mercy Hospital, Kansas City; CNLP, clinical natural language processing; CNV, copy number variant; CSER, Clinical Sequence Evidence-Generating Research; CUMC, Columbia University Medical Center; DDD, Deciphering Developmental Disorders national study; DSD, disorder of sex development; FORGE, Finding Of Rare Disease Genes; GEMINI, Genomic Medicine in Ill Neonates and Infants; GeneDx, GeneDx clinical reference laboratory; GHA, Genomics Health Alliance (12 hospitals and 2 laboratories); GINA, Genetic Information Nondiscrimination Act; GMCK-RD, Genomic Medicine Center Karolinska-Rare Diseases; GUARDIAN, Genomic Uniform-screening Against Rare Diseases in All Newborns; h, hours; HPO, human phenotype ontology; IBD, inflammatory bowel disease; ICU, intensive care unit; INDELs, insertions and deletions; IQR, inter-quartile range; JHU, Johns Hopkins University; KUH, Karolinska University Hospital; MCA, multiple congenital abnormalities; MGHA, Melbourne Genomics Health Alliance; NACS, North Alabama Children’s Specialists, Huntsville; NBS, newborn screening; NEJM, New England Journal of Medicine; NGS, next-generation sequencing; NHS-CUS, National Health Service Cambridge University Hospitals; NICU, neonatal intensive care unit; NSIGHT1, Newborn Sequencing In Genomic Medicine and Public Health-1; NSIGHT2, Newborn Sequencing In Genomic Medicine and Public Health-2; OMIM, Online Mendelian Inheritance in Man; PCR, polymerase chain reaction; PICU, pediatric intensive care unit; PNAS, Proceedings of the National Academy of Sciences USA; RCH, Royal Children’s Hospital, Melbourne; RCHSD, Rady Children’s Hospital San Diego; RCT, randomized control trial; rWES, rapid WES; rWGS, rapid WGS; RUMC, Radboud University Medical Center; S., South; SCH, Seattle Children’s Hospital; SD, standard deviation; SNP, single-nucleotide polymorphism; SNP, single-nucleotide polymorphism; SNVs, single-nucleotide variants; SOC, standard of care; STM, Science Translational Medicine; SV, structural variant; TASMC, Tel-Aviv Sourasky Medical Center; TAT, turnaround time; TCH, Texas Children’s Hospital; TSCH, The Sick Children’s Hospital, Toronto; UCL, UCL Great Ormond Street Institute of Child Health, London; UCLA, University of California at Los Angeles; U of R, University of Rostock; UMC, University Medical Center, Groningen; USD, U.S. Dollar; VUS, variants of uncertain significance; WES, whole-exome sequencing; WGS, whole-genome sequencing; WMU, Wroclaw Medical University; WTC, Wellcome Trust Center for Human Genetics, University of Oxford.
